# Enhanced mitochondrial respiration in peripheral blood mononuclear cells (PBMCs) from young children with overweight/obesity and insulin resistance

**DOI:** 10.1111/eci.70090

**Published:** 2025-06-17

**Authors:** Eugenia Carvalho, Reid D. Landes, Matthew Cotter, Leanna M. Delhey, Elisabet Børsheim, Shannon Rose

**Affiliations:** ^1^ CNC UC ‐ Centre for Neuroscience and Cell Biology University of Coimbra Coimbra Portugal; ^2^ CIBB ‐ Center for Innovative Biomedicine and Biotechnology University of Coimbra Coimbra Portugal; ^3^ Institute for Interdisciplinary Research University of Coimbra Coimbra Portugal; ^4^ University of Arkansas for Medical Sciences, College of Medicine Department of Biostatistics Little Rock Arkansas USA; ^5^ Arkansas Children's Nutrition Center Little Rock Arkansas USA; ^6^ Arkansas Children's Research Institute Little Rock Arkansas USA; ^7^ Department of Epidemiology University of Michigan School of Public Health Ann Arbor Michigan USA; ^8^ University of Arkansas for Medical Sciences, College of Medicine Department of Pediatrics Little Rock Arkansas USA; ^9^ University of Arkansas for Medical Sciences, College of Medicine Department of Geriatrics Little Rock Arkansas USA; ^10^ Department of Health Promotion and Disease Prevention University of Tennessee Health Science Center, College of Nursing Memphis Tennessee USA

**Keywords:** bioenergetics, circulating cells, inflammation, insulin resistance, metabolic health, paediatric obesity

## Abstract

**Background:**

Studies implicating dysfunctional mitochondrial respiration in metabolic tissues in the development of insulin resistance in obesity have only included adults. Peripheral blood mononuclear cells (PBMCs) and platelets have been found to reflect systemic mitochondrial fitness and bioenergetic health. We sought to identify bioenergetic differences in PBMCs and platelets from children with obesity and insulin resistance and determine associations with whole‐body metabolism and/or biomarkers of metabolic health and inflammation.

**Methods:**

We stratified prepubertal children (ages 5–10 years) into three groups: normal weight insulin sensitive (N‐IS; *n* = 20), overweight/obese insulin sensitive (O‐IS; *n* = 28) and overweight/obese insulin resistant (O‐IR; *n* = 17). We measured oxygen consumption rate and proton efflux rate in PBMCs and platelets. We estimated whole‐body resting metabolic rate by bioimpedance and dietary fatty acid oxidation by oral deuterated palmitate and quantifying recovery of D_2_O in urine. We used ANOVA for comparisons among groups and Spearman correlations for associations between circulating cell bioenergetics and whole‐body metabolism and biomarkers.

**Results:**

O‐IS and O‐IR PBMCs exhibited increased maximal mitochondrial respiration and spare respiratory capacity compared to N‐IS. Bioenergetics shifted towards glycolysis in O‐IS PBMCs as compared to both N‐IS and O‐IR PBMCs. In platelets, glycolysis and ATP production rates were decreased in O‐IR compared to O‐IS children. PBMC respiration positively correlated with BMIz, HOMA‐IR and fasting glucose and insulin, but negatively correlated with inflammatory cytokines. Dietary fatty acid oxidation was higher in O‐IS compared to N‐IS children and positively correlated with PBMC spare respiratory capacity. Resting metabolic rate correlated positively with several parameters of PBMC mitochondrial respiration.

**Conclusions:**

PBMCs from young children with overweight/obesity exhibit adaptations to the metabolic stressors associated with insulin resistance, and PBMC metabolism correlates well with whole‐body metabolism.

## Graphical abstract

We investigated measures of mitochondrial function in certain types of blood cells and metabolism in 65 children, 5–10 years old. Compared to children of normal weight (*n* = 20), children with overweight/obesity (*n* = 28) and especially those with insulin resistance (*n* = 17), differed in how their cells produced energy. Differences were linked with body fat, blood glucose and inflammation. This suggests that blood cell metabolism may reflect whole‐body health and help identify early signs of metabolic disease in kids. Created in BioRender. Borsheim, E. (2025). https://BioRender.com/zft3pvn

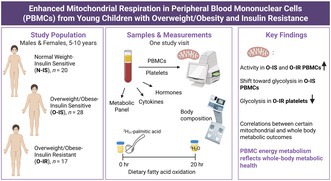

## 1 INTRODUCTION

The dramatic increase in the prevalence of childhood obesity has enormous public health implications and may result in a reduced life span of the current generation of children.^1^ Attributed to the increasing prevalence of childhood obesity, type 2 diabetes (T2D) is being diagnosed in a growing number of young children.^2^ Obesity in childhood puts children at an increased risk of obesity in adulthood as well as severe health complications associated with obesity such as T2D, hypertension, cardiovascular disease, retinopathy and neuropathy, and the risk increases as the age of onset decreases.^3^

Mitochondrial dysfunction in key metabolic tissues including skeletal muscle, the primary site for glucose uptake, has been implicated in the development of insulin resistance and T2D.^4^, ^5^, ^6^ Numerous studies of skeletal muscle biopsies from adults with obesity report not only reduced activities of key mitochondrial enzymes including citrate synthase, malate dehydrogenase, NADH oxidase, succinate oxidase and cytochrome oxidase, but also that reduced activity of these enzymes correlates with measures of insulin resistance.^7^, ^8^, ^9^, ^10^, ^11^, ^12^ Due in part to the invasive nature of tissue biopsies, such studies have not been conducted in paediatric populations, and we know little about mitochondrial function in children with obesity irrespective of insulin resistance or T2D. Studies using post‐exercise phosphocreatine recovery (PCr), a disputed index of muscle mitochondrial oxidative phosphorylation,^13^ indicate muscle mitochondrial dysfunction in adolescents with obesity and insulin resistance.^14^, ^15^

Circulating blood cells including platelets and peripheral blood mononuclear cells (PBMCs), which are composed of lymphocytes and monocytes, are an easily obtainable source to examine bioenergetics and would be a noninvasive alternative to collecting tissue biopsies and have been studied as a measure of systemic mitochondrial fitness, physical function and inflammation.^16^, ^17^ In the context of obesity, Nijhawan et al. reported increases in mitochondrial respiration of circulating monocytes in adults with obesity after undergoing bariatric surgery and that post‐surgery increases in monocyte basal and maximal respiration correlated with increases in skeletal muscle maximal respiration.^18^ Capkova et al. compared mitochondrial enzymatic function of circulating lymphocytes from subjects with normal weight and obesity ranging in age from 17 to 75 years and found decreased activities of cytochrome oxidase and citrate synthase in lymphocytes from subjects with obesity, with the greatest decrease in the youngest (<45 years) and oldest (>65 years) subjects.^19^ In a recent study of mitochondrial respiration of PBMCs from college‐age young adults, Shirakawa et al. reported increased complex I + II linked oxidative capacity, maximal oxidative capacity and increased complex IV capacity in subjects with obesity and fatty liver compared to controls and that enhanced PBMC mitochondrial respiration positively associated with fatty liver scores, fasting insulin, HOMA‐IR and inflammatory markers (CRP, TNFa and IL‐6).^20^ Most recently, we examined PBMC bioenergetics for the first time in a paediatric population.^21^ In a cohort of children and adolescents aged 5–17 years spanning the spectrums of obesity, insulin resistance and glucose intolerance, we found that the ratio of mitochondrial respiration to glycolysis positively associated with BMIz and negatively associated with HbA1c.^21^ Still, no such studies have been done in children at the earliest stages of obesity, prior to the development of insulin resistance, glucose intolerance and other co‐morbidities such as fatty liver.

Our lack of understanding of how mitochondrial dysfunction contributes to the progression from a metabolically healthy state to insulin resistance, glucose intolerance and eventually T2D in childhood obesity impedes the development of much needed treatment strategies aimed at averting the progression into more serious health concerns. The primary aim of this study was to determine whether the bioenergetics of PBMCs and platelets, two easily obtainable populations of circulating cells, differ among young prepubertal children with overweight/obesity, with and without insulin resistance, and children with healthy normal weight. To determine how circulating cell bioenergetics relate to whole‐body metabolism and metabolic health, we further examined correlations between PBMC and platelet bioenergetics with estimated resting metabolic rate (RMR) and whole‐body dietary fatty acid oxidation (FAO), as well as with biomarkers of metabolic health and inflammation.

## 2 METHODS

### 2.1 Study participants

A cohort of 65 prepubertal children were recruited by local advertisements from the Center for Obesity and its Consequences in Health (COACH) clinic and the Endocrinology and Diabetes clinic at Arkansas Children's Hospital. Study inclusion criteria were age 5–10 years at the date of the visit. Exclusion criteria were the presence of known chronic illnesses/disorders (e.g. type 1 diabetes mellitus, neurologic, developmental, endocrine, hepatic, autoimmune, cardiac and renal disorders), prescribed any of the following medications: antipsychotics, thyroid hormone replacement therapy, inhalation/oral steroids, insulin, anabolic drugs and stimulants or being classified as underweight (BMI <5th percentile).

Advertisements were also posted in the community via social media, local newsletters and distribution of flyers through local fairs and events, schools, recreational centres, churches and other local healthcare providers. In addition, participants of previous research studies conducted at Arkansas Children's who indicated willingness to be contacted for future research were also recruited. Participant recruitment was performed after approval by the Institutional Review Board (IRB) at the University of Arkansas for Medical Sciences. The study was registered at ClinicalTrials.gov (NCT03323294). The parents or legal guardians of the study participants gave written informed consent and children older than 7 years of age gave assent to participate.

### 2.2 Study visit

Participants completed a single study visit, taking place early in the morning at the Pediatric Clinical Research Unit (PCRU) at Arkansas Children's Hospital. They arrived at the study site after an overnight fast (no food or drinks except water after midnight). Following consent/assent, and confirmation of inclusion/exclusion criteria, we collected demographic (sex, age, race and ethnicity) and anthropometric data as previously described.^22^ Briefly, body mass (kg) was measured using a calibrated Avery Berkel HL122 Series Platform Scale (Dynamic Scales, Terre Haute, IN, USA). Height (cm) was measured using a stadiometer (Novel Products, Rockton, IL, USA). We calculated BMI from body mass and height as kg/m^2^ and then calculated BMI z‐scores according to CDC using https://cpeg‐gcep.shinyapps.io/quickZ_CDC/. Heart rate (bpm) and systolic and diastolic blood pressure (mmHg) were measured following standard procedures using, a GE Carescape V100 Dinamap vital signs monitor. SBP and DBP were converted to height, age and sex‐adjusted percentiles, using the Canadian Pediatric Endocrine Group's calculator (https://cpeg‐gcep.shinyapps.io/BPz_cpeg/) and then into z‐scores using the R function qnorm for each participant. Following assessment of RMR and body composition, subjects provided blood and urine samples and ingested the stable isotope for whole‐body dietary FAO assessment (details of these methods follow).

### 2.3 Resting metabolic rate (RMR)

We estimated RMR from bioelectrical impedance analysis using the Tanita Body Composition Analyzer Goal Setter TBF‐410 (TANITA Corporation, Arlington Heights, IL, USA). After emptying their pockets, removing any jewellery, jackets, socks and shoes, participants wiped the bottoms of their feet with alcohol pads. Participants then stepped on the scale and were asked to stand still with each foot on a metal pad on the scale, with body weight evenly distributed between both feet, arms hanging freely by the sides of the body and with palms facing the thighs and to look straight ahead during the recording. In addition to recording the estimated RMR (kcal/day), we also recorded the estimated fat mass (kg) and fat‐free mass (kg).

### 2.4 Whole‐body dietary fatty acid oxidation (FAO)

Whole‐body dietary FAO rate was assessed using orally administered deuterated palmitate and measuring the recovery of deuterated water in urine by isotope‐ratio mass spectrometry (IRMS).^23^ After providing a baseline urine sample, fasted participants ingested the isotopically labelled tracer, ^2^H_31_‐palmitic acid (Cambridge Isotope Laboratories, Inc., Tewksbury, MA, USA), which was administered at a dose of 15 mg/kg in a spoon of fat‐free yogurt or applesauce. Following ingestion of the deuterated palmitate, a urine sample was collected at home approximately 20 h later. Participants were instructed to store the urine sample at 4°C or on ice until returned to the study site. Urine was filtered through a .22 μm syringe filter and aliquoted into cryovials for storage at −80°C until analysis. Participants were instructed to maintain their normal diet and to refrain from moderate or vigorous physical activity levels during the testing period.

Deuterium enrichment (as atom percent excess, APE) was measured using the H_2_/H_2_O equilibration method on a GasBench II coupled to a Delta V IRMS (Thermo Scientific, Bremen, Germany). Briefly, 200 μL of urine was placed in a 12 mL round bottom vial (Labco Exetainer, Lampeter UK) and a platinum catalyst rod was added. The tubes were capped and flushed with a mixture of 2% H_2_ at a flow of 100 mL/min for 5 min. The tubes were allowed to equilibrate at room temperature for 40 min before injecting into the IRMS. A loop volume of 100 μL was used, and 3 reference peaks and 9 sample peaks were injected for each sample.

Fatty acid oxidation is calculated as the fractional oxidation rate, or the percent of total ^2^H_31_‐palmitic acid oxidized per hour:

$$\mathrm{FAO}\;\left(\%/\mathrm{hr}\right)=\left(\left({\mathrm{APE}}_{\text{T2}}–{\mathrm{APE}}_{\text{T1}}\right)/\left({\mathrm{APE}}_{\text{T2}}\times T_{2}\right)\right)\times \;100$$

Where: APE_T1_ = deuterium atom percent excess enrichment in urine at baseline, APE_T2_ = deuterium atom percent excess enrichment in urine at 20 h post ^2^H_31_‐palmitic acid ingestion and T_2_ = hours between ^2^H_31_‐palmitic acid ingestion and 20‐h urine sample collection.

### 2.5 Diet Records and Physical Activity Questionaires

To account for dietary fat consumption during the testing period, parents of participants were instructed to record all food and drinks ingested over the 20 h between ingesting the tracer and collecting the last urine sample. Diet data were entered into Elizabeth S. Hands and Associates (ESHA) Food Processor® software for analysis of kcal and nutrient content. Participants, with the help of their parents, were also asked to complete the Physical Activity Questionnaire for elementary‐aged children (PAQ‐C), a 10‐item, self‐administered, 7‐day recall questionnaire that measures general moderate to vigorous physical activity levels to estimate the typical amount of physical activity for their child.

### 2.6 Blood collection and processing

For collection of plasma and PBMCs, approximately 15 mL of fasting blood was collected into EDTA‐vacutainers and gently inverted several times, and placed on ice immediately after collection. Within 30 min of collection, blood was centrifuged at 1500 × *g* for 15 min at 4°C to separate plasma, which was transferred into cryovials, flash frozen on dry ice and stored at −80°C for future analyses. For collection of platelets, approximately 5 mL of fasting blood was collected into EDTA‐vacutainers, gently inverted several times and placed on the bench at room temperature. Within 30 min of collection, blood was centrifuged at 200 × *g* for 10 min at room temperature with no brake to separate platelet‐rich plasma. For collection of serum, approximately 5 mL of fasting blood was collected in red‐top serum vacutainers and allowed to clot at room temperature for 30–60 min prior to centrifuging at 1200 × *g* for 15 min at room temperature. Serum was removed and transferred into cryovials, flash frozen on dry ice and stored at −80°C for future analyses.

### 2.7 PBMC and platelet isolation

PBMCs were isolated as previously described.^24^ Briefly, after spinning the blood and removing plasma, room temperature PBMC wash buffer (Ca^+2^/Mg^+2^ free DPBS with .1% BSA and 2 mM EDTA) equal to the volume of removed plasma was added to EDTA vacutainers. Diluted blood was gently mixed by inversion and then layered onto an equal volume of Histopaque 1077 (Sigma‐Aldrich, St. Louis, MO, USA) and centrifuged at 400 × *g* for 30 min at room temperature with no brake. The PBMC layer was transferred into a new conical tube containing 20 mL PBMC wash buffer. PBMCs were washed two times with 20 mL PBMC wash buffer and collected by centrifuging at 250 × *g* for 10 min. Contaminating red blood cells were lysed with a brief (~10 s) incubation with 1 mL ice‐cold water. Viable PBMCs were counted using a haemocytometer and trypan blue exclusion. Platelets were isolated as previously described.^24^ Platelet‐rich plasma was supplemented with 10% (v/v) 100 mM EGTA in PBS and centrifuged at 1000 × *g* for 10 min with no brake. Platelets were gently washed in 10 mM EGTA in PBS and centrifuged at 1000 × *g* for 10 min with no brake, gently re‐suspended in 10 mM EGTA in PBS and counted using optical density by measuring absorbance at 800 nm using a spectrophotometer (Shimadzu, Kyoto, Japan) as described by Walkowiak et al.^25^

### 2.8 Extracellular flux (Seahorse) analysis

Freshly isolated PBMCs and platelets were plated on poly‐d‐lysine coated XF‐96 well plates at a density of 2.5e5 and 10e6 cells per well, respectively, in Seahorse XF RPMI without phenol red supplemented with 2 mM glutamine, 1 mM pyruvate, 11 mM glucose and 1 mM HEPES (all from Agilent Technologies, Santa Clara, CA, USA). Inhibitors/uncouplers were obtained from Sigma‐Aldrich and used at the following final concentrations: 1 μM oligomycin, 1.5 μM 2‐[2‐[4(trifluoromethoxy)phenyl] hydrazinylidene]‐propanedinitrile (FCCP), 1 μM antimycin A, 1 μM rotenone and 50 mM 2‐deoxy‐d‐glucose (2‐DG). We used three injection strategies to interrogate cellular bioenergetics. For the mitochondrial stress test, oligomycin, FCCP and a mixture of rotenone and antimycin A were injected sequentially. For the glycolytic rate assay, the antimycin A/rotenone mixture was injected first, followed by 2‐DG. Lastly, for the ATP production rate assay, oligomycin was injected, followed by antimycin A/rotenone. Oxygen consumption rates (OCR; pmol O_2_/min) and proton efflux rates (PER; pmol H^+^/min) were determined over 3‐min increments. From the mitochondrial stress test, basal respiration, maximal respiration and spare respiratory capacity, in OCR units, were calculated. Maximal respiration is the maximal oxygen consumption rate attained by adding the uncoupler FCCP, which stimulates the respiratory chain to operate at maximum capacity. Spare respiratory capacity (maximal respiration minus basal respiration) indicates the capability of the cell to respond to an energetic demand as well as how closely the cell is to respiring at its theoretical maximum. From the glycolytic rate assay, basal glycolysis in PER units and the relative ratio of basal mitochondrial respiration to glycolysis (OCR/PER) were calculated. From the ATP production rate assay, mitochondrial ATP production rate (mitoATP) and glycolysis ATP production rate (glycoATP) were calculated, as well as the total ATP production rate (mitoATP plus glycoATP), all in units of pmol ATP/min. Because cells were counted immediately before the assay and equal numbers of viable cells were plated per well, Seahorse data are internally normalized to cell count. Due to missing samples and because this study started prior to the release of the ATP production rate assay, not all Seahorse assays were able to be performed in the maximal number of participants.

### 2.9 Blood biochemical analyses

Plasma insulin was analysed by electrochemical luminescence according to the manufacturer's protocol (Meso Scale Discovery, Rockville, MD). Serum glucose was analysed using an YSI 2900 biochemistry analyzer (YSI Life Sciences, Yellow Springs, OH). Serum total cholesterol, high density lipoprotein cholesterol (HDL‐C), low density lipoprotein cholesterol (LDL‐C), triglycerides (TGs), glycerol, lactate and C‐reactive protein (CRP), were all analysed on an RX Daytona Clinical Analyzer (Randox, Kearneysville, WV).

HOMA‐IR was calculated from fasting insulin (μIU/mL) and glucose (mg/dL) using the following equation: HOMA‐IR = (glucose × insulin)/405.^26^

### 2.10 Cytokine analyses

Leptin, IL‐6, IL‐8, MCP‐1, TNFα and IL‐1β concentrations were determined in plasma by multiplex immunoassays. Specifically, the MILLIPLEX MAP Human Adipokine Magnetic Bead Panel 2 (Millipore Sigma, USA) was used following the manufacturer's instructions, and samples were analysed on a Luminex® 200™ running xPONENT® software. Adiponectin was quantified in plasma by ELISA using the Human Adiponectin ELISA (Millipore Sigma) following the manufacturer's instructions.

### 2.11 Statistical analyses

Study participants were stratified based on both BMI and HOMA‐IR. Study participants were classified as normal weight if their age‐ and sex‐adjusted BMI was below the 85th percentile, and as overweight/obese if their age‐ and sex‐adjusted BMI was at or above the 85th percentile. Participants were further classified as IR if their HOMA‐IR was ≥1.95.^27^ Combinations of these two classifications (BMIz and HOMA‐IR) produced three groups of participants: normal weight and insulin sensitive (N‐IS), overweight/obese and insulin sensitive (O‐IS) and overweight/obese and insulin resistant (O‐IR). There were no participants who were of normal weight and were IR.

Means and standard deviations (SD) summarized continuous measures and percentages summarized categorical measures. To address the primary aim of the study, we used a one‐factor analysis of variance (ANOVA) with metabolic group (N‐IS, O‐IS and O‐IR) as the factor, and allowed each group to have its own variance, thus a linear mixed model or Welch's ANOVA. Error degrees of freedom were estimated with Kenward‐Roger method.^28^ Pairwise comparisons of N‐IS to each of O‐IS and O‐IR were made with contrasts (i.e. ‘post hoc tests’) within the model. We checked normal assumptions using the residuals from the model, and when these assumptions were violated, used Wilcoxon signed‐rank tests to make pairwise comparisons. We considered the Wilcoxon signed rank tests and *t*‐tests to corroborate each other when their *p*‐values were within ±.05 of each other; otherwise, we report the Wilcoxon signed‐rank test as primary. Because of the multiple comparisons and testing, we estimated the positive False Discovery Rate (pFDR) from the ANOVA results using algorithm 3 in Storey.^29^ For the secondary aim of the study, we computed Spearman (rank) correlations for the bioenergetic parameters paired with metabolic health and inflammation parameters. Results with a *p* < .05 were considered statistically significant. All ANOVA and correlational analyses were conducted in SAS/STAT software, version 9.4 for Windows (SAS Institute); the pFDR was estimated in R V4.2.2 with custom code. Figures were made using Prism v9 (Graph Pad). The de‐identified data used to produce the results for this work and SAS code are included in the Appendix S1.

### 2.12 Sample size considerations

This study was originally planned to have 15 participants in each of the three groups for a total of 45 participants. This would have allowed us to detect a difference in means between a pair of groups of size 1.05 SD with .80 power on a .05 level test, where SD is the within‐group SD assumed equal among the groups. However, we were able to recruit an additional 20 participants (65 in total); hence the detectable difference between groups was lower than the 1.05 SD. Since the group sizes were unbalanced and the group SDs were assumed heterogeneous in the analyses, we did not update the detectable effect size.

## 3 RESULTS

We provide summary statistics of subject characteristics in Table 1. Groups were well balanced with respect to age, sex and ethnicity, and by design, they differed in terms of BMI‐z and HOMA‐IR. Table 1 does not provide statistical comparisons among the groups because we had no a priori hypotheses, and because the characteristics BMIz (a function of sex, age, weight and height) and HOMA‐IR (a function of insulin and glucose) were used to define the groups. We note that HOMA‐IR differences among the groups were driven mostly by differences among fasting insulin levels, as fasting glucose had less variability among the groups. In this young cohort, all participants had normal fasting glucose.

**TABLE 1:** Cohort characteristics.

Outcome	Overall	N‐IS	O‐IS	O‐IR
	(*n* = 65)	(*n* = 20)	(*n* = 28)	(*n* = 17)
Age (years)	7.2 ± 1.4	6.8 ± 1.2	7.1 ± 1.4	7.9 ± 1.4
Male (%)	35 (54%)	14 (70%)	14 (50%)	7 (41%)
Non‐Hispanic Caucasian (%)	33 (51%)	13 (65%)	14 (50%)	6 (35%)
Height (cm)	129.3 ± 11.2	124.3 ± 8.5	128.4 ± 11.2	136.8 ± 10.5
Weight (kg)	35.7 ± 12.8	24.6 ± 4.4	34.9 ± 9.1	50.0 ± 10.9
Waist Circumference (cm)	64.6 ± 12.8	54.4 ± 4.2	63.6 ± 10.1	78.4 ± 11.4
BMI‐z score	1.46 ± 1.18	.08 ± .67	1.81 ± .55	2.50 ± .90
SBP‐z score	.83 ± 1.00	.32 ± 1.01	.89 ± 1.02	1.36 ± .61
DBP‐z score	.34 ± .82	.16 ± .90	.38 ± .83	.50 ± .72
Glucose (mg/dL)	90.9 ± 10.5	88.7 ± 6.35	89.4 ± 9.38	96.0 ± 14.4
Insulin (μIU/mL)	8.18 ± 7.98	3.65 ± 1.55	5.60 ± 1.91	17.8 ± 10.5
HOMA‐IR	1.91 ± 2.16	.80 ± .36	1.22 ± .38	4.34 ± 3.11
Total Cholesterol (mg/dL)	156 ± 31	148 ± 27	159 ± 34	163 ± 30
HDL‐C (mmol/L)	1.36 ± .29	1.45 ± .30	1.41 ± .29	1.14 ± .16
LDL‐C (mmol/L)	2.36 ± .73	2.12 ± .61	2.37 ± .76	2.63 ± .74
Triglycerides (mmol/L)	.72 ± .42	.56 ± .19	.72 ± .54	.93 ± .35
Glycerol (μg/mL)	92 ± 33	94 ± 40	87 ± 31	99 ± 30
Lactate (mmol/L)	2.17 ± .57	2.07 ± 057	2.07 ± .46	2.48 ± .66
CRP (mg/L)	1.47 ± 2.27	.24 ± .18	1.66 ± 2.22	2.79 ± 3.16
Leptin (ng/mL)	767 ± 787	101 ± 88	778 ± 643	1495 ± 792
Adiponectin (ng/mL)	13,439 ± 5569	15,198 ± 5270	14,778 ± 5488	9167 ± 3721
Leptin/Adiponectin	.085 ± .106	.007 ± .007	.071 ± .078	.195 ± .117
IL6 (pg/mL)	44 ± 104	52 ± 92	53 ± 135	22 ± 43
MCP1 (pg/mL)	123 ± 37	128 ± 37	120 ± 39	123 ± 34
TNFα (pg/mL)	6.6 ± 6.7	8.5 ± 8.4	7.1 ± 6.8	3.8 ± 1.9
IL1β (pg/mL)	2.3 ± 6.0	2.7 ± 6.4	2.8 ± 7.2	.9 ± .8
IL8 (pg/mL)	11.6 ± 15.4	12.3 ± 15.1	12.2 ± 18	8.5 ± 7.9

*Note*: The data are expressed as means ± standard deviations or *n* (%).

Abbreviations: BMI, body mass index; CRP, C reactive protein; DBP, diastolic blood pressure; HDL‐C, high‐density lipoprotein cholesterol; HOMA‐IR, homeostatic model assessment for insulin resistance; IL1β, interleukin 1 beta; IL‐6, interleukin 6; IL‐8, interleukin 8; LDL‐C, low‐density lipoprotein cholesterol; MCP1, monocyte chemoattractant protein 1; SBP, systolic blood pressure; TNFα, tumour necrosis factor alpha.

### 3.1 PBMC bioenergetics

**FIGURE 1:**
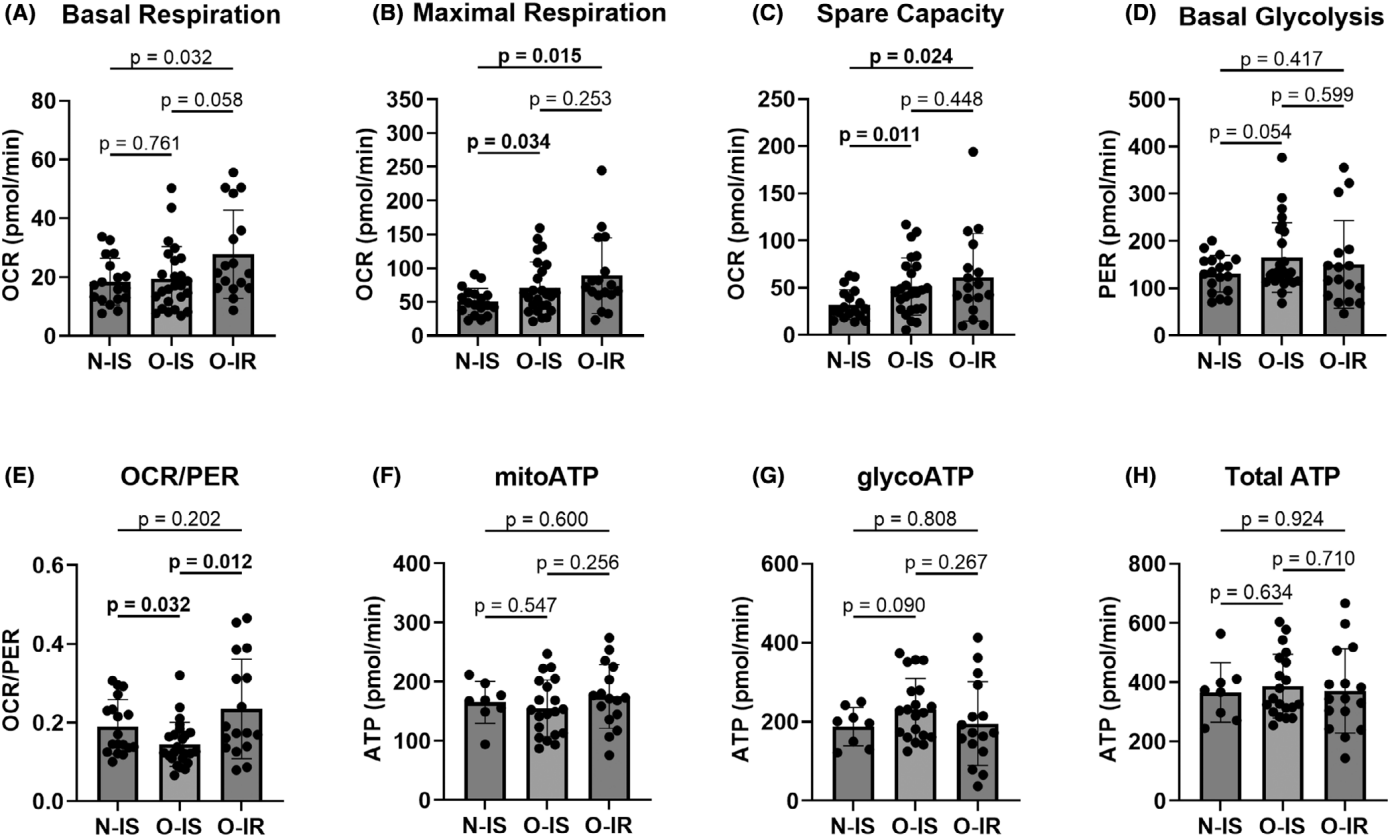
Peripheral blood mononucler cell (PBMC) Bioenergetics. (A) Basal respiration in pmolO_2_/min; (B) Maximal respiration in pmolO_2_/min; (C) Spare capacity in pmolO_2_/min; (D) Basal glycolysis, measured in pmolH^+^/min; (E) OCR/PER; (F) mitoATP production rate in pmolATP/min; (G) glycoATP productionr ate in pmolATP/min; and (H) Total ATP production rate in pmolATP/min. Normal weight insulin sensitive (N‐IS); Overweight/obese insulin sensitive (O‐IS); Overweight/obese insulin resistant (O‐IR). For A–E, *n* = 25 N‐IS, *n* = 17 O‐IS and *n* = 17 O‐IR. For F–H, *n* = 20 N‐IS, *n* = 16 O‐IS and *n* = 16 O‐IR. Seahorse assays were conducted in freshly isolated PBMCs plated at 250,000 cells/well. Results with a *p* < .05 were considered statistically significant and are indicated with bold font. ATP, adenosine triphosphate; glycoATP, rate of ATP produced by glycolysis; mitoATP, rate of ATP produced by mitochondrial respiration; OCR/PER, ratio of oxygen consumption rate to proton efflux rate (ratio of mitochondrial respiration to glycolysis); PBMC, peripheral blood mononuclear cells.

PBMC basal respiration was ~50% higher in O‐IR compared to N‐IS children (Figure 1A). Maximal respiration was 40% and 75% higher in O‐IS and O‐IR as compared to N‐IS children (Figure 1B) while spare respiratory capacity was 60% and 90% higher in O‐IS and O‐IR as compared to N‐IS children (Figure 1C). Basal glycolysis rates were not significantly different between groups (Figure 1D); however, OCR/PER, a ratio of mitochondrial respiration to glycolysis, was significantly lower (e.g. shifted towards glycolysis) in PBMCs from O‐IS as compared to both N‐IS and O‐IR children (Figure 1E). The rates of ATP production from either mitochondrial respiration or glycolysis did not differ significantly between groups (Figure 1F–H).

### 3.2 Platelet bioenergetics

**FIGURE 2:**
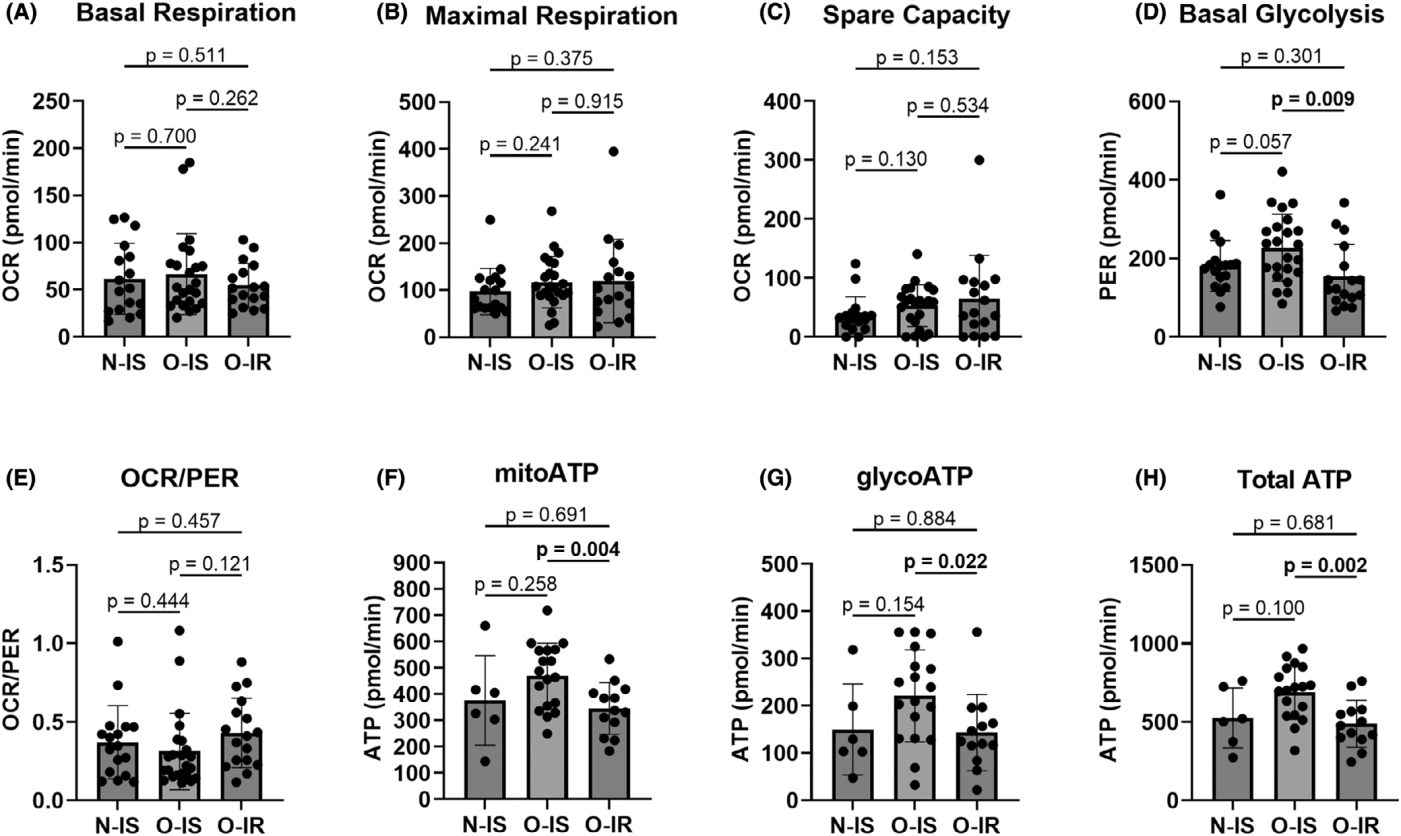
Platelet bioenergetics. (A) Basal respiration in pmolO_2_/min; (B) Maximal respiration in pmolO_2_/min; (C) Spare capacity in pmolO_2_/min; (D) Basal glycolysisin pmolH^+^/min; (E) OCR/PER; (F) mitoATP production rate in pmolATP/min; (G) glycoATP production rate in pmolATP/min; and (H) Total ATP production rate in pmolATP/min. Normal weight insulin sensitive (N‐IS); Overweight/obese insulin sensitive (O‐IS); Overweight/obese insulin resistant (O‐IR). For A–E, *n* = 23 N‐IS, *n* = 17 O‐IS and *n* = 17 O‐IR. For F–H, *n* = 18 N‐IS, *n* = 13 O‐IS and *n* = 13 O‐IR. Seahorse assays were conducted in freshly isolated platelets plated at 10 million cells/well. Results with a *p* < .05 were considered statistically significant and are indicated with bold font. ATP, adenosine triphosphate; glycoATP, rate of ATP produced by glycolysis; mitoATP, rate of ATP produced by mitochondrial respiration; OCR/PER, ratio of oxygen consumption rate to proton efflux rate (ratio of mitochondrial respiration to glycolysis).

When platelet respiration was interrogated using the mitochondrial stress test, all parameters of mitochondrial respiration were similar between groups (Figure 2A–C). While lower rates of glycolysis in platelets from O‐IR as compared to O‐IS children were observed (Figure 2D), OCR/ECAR did not differ among groups (Figure 2E). ATP production rates from both mitochondrial oxidative phosphorylation and glycolysis were ~30% lower in platelets from O‐IR as compared to O‐IS participants (Figure 2F–H).

### 3.3 Whole‐body metabolism

Results of the RMR and dietary FAO are presented in Table 2. Total RMR differed among all three groups, with the lowest RMR in the N‐IS group and the highest RMR in the O‐IR group. Since RMR is dependent on body weight and composition, we also used an ANCOVA with fat‐free mass as a covariate and compared the means. In this additional analysis, an increase of 1 kg in fat‐free mass was estimated to result in a 31 kcal/day increase in RMR; however, the ordering of the three groups' means was the same and the groups still statistically differed. The dietary FAO rate of the O‐IS group was statistically higher, by 1.4 times, than that of the N‐IS group; however, when adjusted for fat‐free mass, no differences in dietary FAO were found among the groups. Because oxidation of the ingested deuterated palmitate may be influenced by a participant's diet consumed during the testing period, all participants logged their food and drink for the duration of the testing period (~20 h). Presented in Table 2, intake of total protein, carbohydrates, fats and total calories did not differ among groups. We also tested for differences in general levels of physical activity between the groups as this may also impact fatty acid oxidation and based on scores from the Physical Activity Questionnaire for elementary school‐aged children (PAQ‐C), we did not detect differences between groups in terms of regular physical activity levels (Table 2).

**TABLE 2:** Whole‐body metabolism.

Variable	N‐IS	O‐IS	O‐IR
	(*n* = 18)	(*n* = 28)	(*n* = 16)
RMR (kcal/day/kg body weight)	42.67 (3.79)^a^	35.68 (3.91)^b^	30.19 (317)^c^
Dietary FAO (%/h)	.31 (.16)^a^	.44 (.24)^b^	.39 (.24)^ab^
Fat intake (g/day)	67 (36)^a^	78 (40)^a^	64 (25)^a^
Protein intake (g/day)	63 (28)^a^	67 (35)^a^	61 (18)^a^
Carbohydrate intake (g/day)	206 (79)^a^	243 (107)^a^	232 (115)^a^
Energy intake (kcal/day)	1660 (703)^a^	1920 (825)^a^	1727 (647)^a^
Physical Activity Score	3.03 (.56)^a^	3.00 (.81)^a^	2.68 (.69)^a^

*Note*: The data are expressed as means (standard deviations). Groups with matching superscripts indicate there is *no* statistical difference between the groups at *p* < .05 level.

Abbreviations: FAO, fatty acid oxidation; RMR, resting metabolic rate.

In Figures 1 and 2 and Table 2, we made 69 comparisons, of which 15 (22% of 69) were significant. The estimated pFDR was .062 with an upper 95% confidence bound of .169. This means that of the 15 significant results, the expected number of false discoveries is 1, and we are 95% confident that there are not more than 3 false discoveries.

### 3.4 Correlations between circulating cells and whole‐body metabolism

**FIGURE 3:**
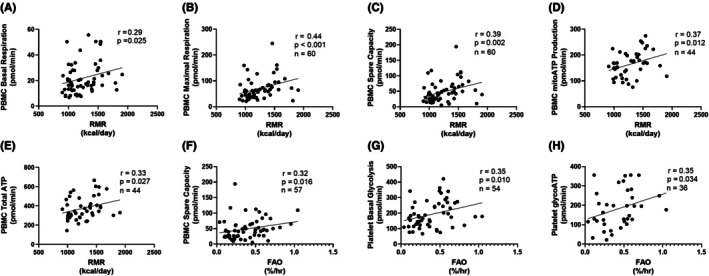
Cellular bioenergetics correlations with resting metabolic rate (RMR) and fatty acid oxidation (FAO). RMR was correlated with (A) Peripheral blood mononuclear cell (PBMC) basal respiration; (B) PBMC maximal respiration; (C) PBMC spare capacity; (D) PBMC mitoATP production rate; (E) PBMC total ATP production rate. FAO was correlated with (F) PBMC spare capacity; (G) Platelet basal glycolysis; (H) Platelet glycoATP production rate. ATP, adenosine triphosphate; FAO, fatty acid oxidation; glycoATP, rate of ATP produced by glycolysis; mitoATP, rate of ATP produced by mitochondrial respiration; OCR/PER, ratio of oxygen consumption rate to proton efflux rate (ratio of mitochondrial respiration to glycolysis); PBMC, peripheral blood mononuclear cells; RMR, resting metabolic rate.

An additional goal of this study was to determine whether RMR and dietary FAO measures relate to PBMC and/or platelet bioenergetics. Bioenergetics parameters measured in PBMCs correlated positively with RMR, including basal respiration, maximal respiration and spare respiratory capacity, and with mitochondrial and total ATP production rates (Figure 3A–E). No significant correlations were found between platelet bioenergetics and RMR. Dietary FAO correlated positively with PBMC spare respiratory capacity (Figure 3F) and platelet basal glycolysis and glycoATP (Figure 3G,H). Correlations not reaching significance are presented in Table 3.

**TABLE 3:** Correlations between whole‐body metabolism and circulating cells bioenergetics parameters.

Variable *X*	Variable *Y*	*N*	CORR	95% CI	*p*
RMR	PBMC basal glycolysis	60	.15	(−.11, .39)	.259
RMR	PBMC OCR/PER	60	.01	(−.24, .26)	.929
RMR	PBMC glycoATP	44	.22	(−.08, .49)	.155
RMR	Platelet basal respiration	57	−.11	(−.36, .16)	.41
RMR	Platelet maximal respiration	57	.02	(−.24, .28)	.872
RMR	Platelet spare capacity	57	.18	(−.08, .42)	.178
RMR	Platelet basal glycolysis	57	−.19	(−.43, .07)	.156
RMR	Platelet OCR/PER	57	.10	(−.16, .35)	.466
RMR	Platelet mitoATP	37	.00	(−.32, .32)	.998
RMR	Platelet glycoATP	37	−.01	(−.33, .32)	.948
RMR	Platelet total ATP	37	.04	(−.29, .36)	.803
FAO	PBMC basal respiration	57	.01	(−.25, .27)	.915
FAO	PBMC maximal respiration	57	.22	(−.04, .45)	.095
FAO	PBMC basal glycolysis	57	.26	(0, .49)	.053
FAO	PBMC OCR/PER	57	−.22	(−.45, .04)	.108
FAO	PBMC mitoATP	43	.14	(−.17, .42)	.365
FAO	PBMC glycoATP	43	.23	(−.08, .5)	.145
FAO	PBMC Total ATP	43	.21	(−.1, .48)	.184
FAO	Platelet basal respiration	54	.02	(−.25, .29)	.895
FAO	Platelet maximal respiration	54	.12	(−.15, .38)	.369
FAO	Platelet spare capacity	54	.23	(−.04, .47)	.101
FAO	Platelet OCR/PER	54	−.24	(−.48, .03)	.086
FAO	Platelet mitoATP	36	.23	(−.11, .52)	.172
FAO	Platelet total ATP	36	.30	(−.03, .57)	.078

Abbreviations: ATP, adenosine triphosphate; FAO, fatty acid oxidation; glycoATP, rate of ATP produced by glycolysis; mitoATP, rate of ATP produced by mitochondrial respiration; OCR/PER, ratio of oxygen consumption rate to proton efflux rate (ratio of mitochondrial respiration to glycolysis); PBMC, peripheral blood mononuclear cells; RMR, resting metabolic rate.

### 3.5 Correlations between circulating cells bioenergetics and markers of metabolic health and inflammation

**FIGURE 4:**
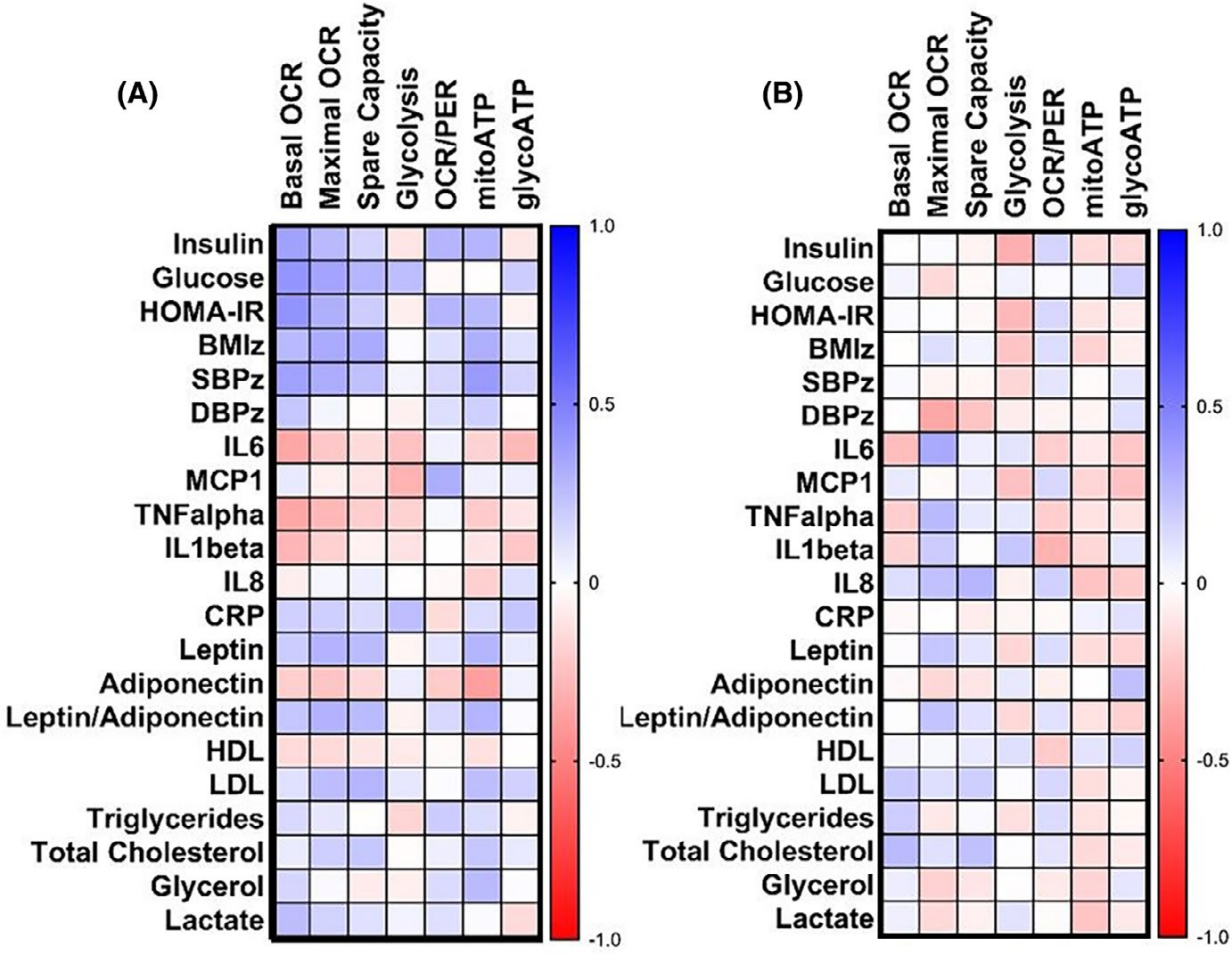
Heatmaps of correlation coefficients between bioenergetics parameters and markers of metabolic health and inflammation for PBMC (A) and Platelets (B). ATP, adenosine triphosphate; BMI, body mass index; CRP, C reactive protein; DBP, diastolic blood pressure; glycoATP, rate of ATP produced by glycolysis; HDL, high‐density lipoprotein cholesterol; HOMA‐IR, homeostatic model assessment for insulin resistance; IL1beta, interleukin 1 beta; IL‐6, interleukin 6; IL‐8, interleukin 8; LDL, low‐density lipoprotein cholesterol; MCP1, monocyte chemoattractant protein 1; mitoATP, rate of ATP produced by mitochondrial respiration; OCR, oxygen consumption rate; OCR/PER, ratio of oxygen consumption rate to proton efflux rate (ratio of mitochondrial respiration to glycolysis); PBMC, peripheral blood mononuclear cells; RMR, resting metabolic rate; SBP, systolic blood pressure; TNF, tumour necrosis factor.

Because we have previously reported measures of metabolic health and inflammation and the differences between N‐IS, O‐IS and O‐IR groups in this cohort,^22^, ^30^ we do not report on those measures here. However, a secondary aim of this study was to determine whether PBMC and/or platelets bioenergetics parameters are related to measures of metabolic health and markers of inflammation. Figure 4 presents correlation matrices for PBMC (4A) and platelet (4B) bioenergetics parameters and metabolic health/inflammatory marker pairs. Similar to the FAO and RMR results, we found numerous associations with bioenergetics parameters measured in PBMCs, but few in platelets. Fasting blood glucose levels positively associated with PBMC basal respiration (*p* < .001), maximal respiration (*p* = .001) and spare capacity (*p* = .007). Insulin and HOMA‐IR both positively associated with PBMC basal respiration (*p*s = .002 and <.001, respectively). BMIz positively associated with PBMC basal respiration (*p* = .014), maximal respiration (*p* = .005) and spare capacity (*p* = .007). For blood pressure, SBPz correlated with PBMC basal respiration (*p* = .004), maximal respiration (*p* = .011) and mitoATP production rate (*p* = .005).

Several PBMC bioenergetics parameters correlated with markers of inflammation. TNFα negatively correlated with PBMC basal and maximal respiration (*p* = .001 and .009, respectively), while IL‐6 negatively correlated with basal respiration (*p* = .004). MCP‐1 negatively correlated with PBMC glycolysis (*p* = .008) and positively correlated with OCR/PER (*p* = .005). The adipokine leptin correlated positively with PBMC maximal respiration (*p* = .011) while adiponectin negatively correlated with mitoATP production rate (*p* = .008) and the ratio of leptin to adiponectin positively correlated with PBMC maximal respiration (*p* = .012). Among lipids, positive correlations were found between LDL and PBMC maximal (*p* = .037) and spare capacity (*p* = .026). Interestingly, lactate positively correlated with basal respiration in PBMCs (*p* = .038).

Fewer platelet bioenergetics parameters correlated with markers of metabolic health. Insulin and HOMA‐IR both correlated negatively with platelet glycolysis (*p*s = .017 and .038, respectively) while DBPz negatively correlated with platelet spare respiratory capacity (*p* = .009). Among markers of inflammation, IL6 and TNFα were found to correlate positively with platelet spare capacity respiration (*p*s = .010 and .040, respectively), while IL1β was negatively correlated with platelet OCR/PER (*p* = .039). Among the lipids measured, total cholesterol correlated positively with platelet basal respiration (*p* = .047).

In Figures 3 and 4 and Table 3, we made 326 hypothesis tests, of which 45 (13.8% of 326) were significant. The estimated pFDR was .262 with an upper 95% confidence bound of .358. This means that of the 45 significant results, the expected number of false discoveries is 12, and we are 95% confident that there are not more than 16 false discoveries.

## 4 DISCUSSION

Our primary aim for this study was to determine if circulating cell bioenergetics differ among young prepubertal children with and without overweight/obesity and insulin resistance. We also sought to determine the relationships between circulating cell bioenergetics and measures of overall metabolic health and inflammation in young children spanning the spectrums of BMI and insulin sensitivity. We report novel bioenergetic differences in both PBMCs and platelets from insulin resistant children with overweight/obesity, namely elevated mitochondrial respiration in PBMCs and reduced mitochondrial respiration and glycolysis in platelets. Elevations in PBMC mitochondrial respiration correlated with increases in RMR and dietary FAO and numerous other measures of metabolic health and inflammation.

### 4.1 PBMC and platelet bioenergetics

Our bioenergetics analyses uncovered elevations in maximal (uncoupled) mitochondrial respiration and the spare respiratory capacity in PBMCs from young children with overweight/obesity, with even larger elevations in those children with insulin resistance. The ratio of mitochondrial respiration to glycolysis was shifted towards glycolysis in PBMCs from insulin sensitive children with overweight/obesity compared to those with normal weight. However, we found a shift in the opposite direction (towards mitochondrial respiration) in PBMCs from insulin resistant children with overweight/obesity, as compared to insulin sensitive children with overweight/obesity. Similarly, in our recent study of children and adolescents ages 5–17 years who spanned the spectrums of obesity, insulin resistance and glucose intolerance, we used multiple linear regression to test whether PBMC OCR/ECAR associated with BMIz, HOMA‐IR and HbA1C and found that PBMC OCR/ECAR positively associated with BMIz but negatively associated with HbA1c (1). Put simply at higher BMIs, metabolism of circulating PBMCs shifted away from glycolysis and towards mitochondrial respiration, but as glycemic control declined, metabolism shifted back towards glycolysis. In that study, we did not detect a significant association between HOMA‐IR and PBMC metabolism, which may be due to the differences between cohorts including a wider age range and an overall older cohort (median age of 10 years) with the onset of puberty in many participants, and HbA1C >5.7 in approximately 20% of participants, and the differences in methods (ANOVA here vs. multiple linear regression controlling for BMIz and HbA1C).

The upregulation of PBMC mitochondrial respiration with overweight/obesity and the bioenergetic shift towards mitochondrial respiration with insulin resistance likely reflects compensatory metabolic responses to chronic overnutrition and to metabolic stressors associated with insulin resistance such as dyslipidemia (e.g. induction of mitochondrial FAO). Supporting this notion, Bohm et al. reported increased mitochondrial respiration associated with increased insulin resistance in a small study of human subcutaneous preadipocytes and differentiated adipocytes derived from metabolically unhealthy (insulin resistant but not diabetic) obese adults compared to metabolically healthy obese adults.^31^ The authors suggest that maintaining this compensatory state of increased respiration to prevent the transition from insulin resistant to T2D.

While not examined here, perhaps whole‐body insulin resistance applies to PBMCs as well, and due to a reduced insulin‐stimulated glucose uptake, these immune cells shift their metabolism towards mitochondrial oxidative phosphorylation. It is important to note the critical role cell metabolism plays in dictating the immune functions of lymphocytes and monocytes, the primary components of PBMCs and the impact of these metabolic shifts on immunity and their role in obesity‐associated inflammation should be studied.

As expected, the bioenergetic profiles of platelets were very different from PBMCs. While platelet bioenergetics did not differ between children with normal weight and overweight/obesity, we observed ~30% reduced glycolysis and mitochondrial ATP production in platelets from insulin resistant as compared to insulin sensitive children with overweight/obesity. The metabolic stressors associated with developing insulin resistance may impair platelet ATP production, which may have implications for clotting and cardiovascular disease risk.

### 4.2 Correlations between RMR and dietary FAO and PBMC and platelet bioenergetics

Two measures of whole‐body metabolism, RMR and dietary FAO, positively correlated with PBMC spare respiratory capacity. Spare respiratory capacity considers both mitochondrial respiration at rest and at maximal capacity, and in PBMCs, this parameter of bioenergetics may track with and relate to whole‐body metabolism. As RMR also correlated with basal and maximal respiration in PBMCs, we conclude that overall PBMC mitochondrial respiration correlates well with RMR. Interestingly, the only relationship we observed with platelet bioenergetics was a positive correlation between platelet glycolysis and dietary FAO. This positive association between glycolysis rates in platelets and whole‐body FAO was surprising but may reflect similarities in responses to varying energy demands.

### 4.3 Correlations between markers of metabolic health/inflammation and circulating cell bioenergetics

Whether circulating cell bioenergetics relates to measures of metabolic health and inflammation is an ongoing area of study in numerous disease areas as circulating cells are exposed to many metabolically important factors in addition to microRNAs and other types of signalling molecules and may be sensitive to changes in such factors and respond metabolically. Overall, we found that PBMCs bioenergetics correlated with numerous metabolic and inflammatory markers, whereas few correlations were found when examining platelet bioenergetics. In this cohort of young children, both insulin levels and HOMA‐IR, an index of insulin resistance that considers fasting glucose and insulin levels, were positively associated with PBMC basal and maximal respiration as well as the ratio of mitochondrial respiration and glycolysis (OCR/PER). Similarly, we found that as fasting glucose levels increased, so did PBMC basal respiration, maximal respiration and basal glycolysis (Figure 4). These findings may simply reflect a metabolic response to increased availability of glucose as a fuel. Positive associations between BMIz and PBMC basal respiration, maximal respiration and mitoATP production rate may also reflect an adaptation to excess nutrient availability. The finding of a positive association between blood lactate levels and PBMC basal respiration is supported by recent work showing lactate serves as a mitochondrial substrate and a signal to the cell to increase mitochondrial ATP production while also suppressing glycolysis, reducing the mitochondrial NADH/NAD+ ratio and suppressing mitochondrial ROS generation.^32^ Providing lactate in culture media was also shown to enhance T‐cell proliferation and effector function, suggesting there are implications for immune function and inflammation that deserve further exploration.^32^

When markers of inflammation were examined, we found that an increase in PBMC basal respiration was associated with an increase in CRP levels while mitoATP production rate positively correlated with the proinflammatory adipokine, leptin and negatively correlated with the anti‐inflammatory adipokine, adiponectin. Three inflammatory cytokines, TNFα, IL‐6 and IL‐1β all negatively correlated with PBMC basal respiration, while MCP‐1, an inflammatory chemokine, negatively correlated with PBMC glycolysis. The bioenergetic shifts in PBMCs in overweight/obesity and insulin resistance may be a protective mechanism to reduce inflammatory cytokine production. As functioning immune cells, the metabolic phenotype of PBMCs plays a major role in activation, differentiation and immune function.^33^, ^34^, ^35^ Given the critical role that inflammation plays in driving obesity‐related metabolic disorders,^36^, ^37^, ^38^ alterations in immune cell metabolism and bioenergetics associated with obesity and its associated metabolic dysfunction and inflammation need to be clearly defined.

### 4.4 Fatty acid oxidation

The estimated oxidation rates of the dietary fatty acid, palmitate, were 1.4 times higher in young children with overweight/obesity who were insulin sensitive, compared to children with normal weight who were insulin sensitive. When adjusting for FFM, no difference between groups could be detected (the difference between the insulin sensitive children with and without overweight/obesity was attenuated to being 1.24 times higher in the overweight/obese group). The literature on whole‐body FAO rates are mixed with some suggesting higher exogenous FAO in kids with obesity.^39^ In our hands, using the same deuterated palmitate technique in toddlers, we found higher rates of oxidation of dietary palmitate in babies who were born to mothers with obesity as compared to babies born to mothers with normal weight, and the higher oxidation was driven by breastfeeding duration.^40^

A major strength of this study is that the young age of the cohort of prepubertal children enabled us to study the relationships among obesity and insulin resistance with PBMC bioenergetics, clinical measures of metabolic health and markers of inflammation at the early stages of obesity and metabolic dysfunction, prior to the onset of glucose intolerance or cardiovascular dysfunction. Furthermore, the influence of puberty on insulin resistance is avoided, as are other lifestyle factors (e.g. smoking) that would influence the measured outcomes. This study also has several limitations. PBMC are comprised mostly of lymphocytes, but are a mixed population of multiple immune cell types; thus, our bioenergetics outcomes could be influenced by altered proportions of immune cells. In certain vulnerable populations, such as children, this limitation will be difficult to overcome due to the large amount of blood required to isolate sufficient numbers of specific immune cell populations required for bioenergetics measures. Immunophenotyping each sample to control for shifts in immune cell populations is one potential alternative but adds significant costs. Previous studies on children have not reported major shifts in the proportions of circulating immune cells with obesity.^41^ Another limitation is the lack of indirect calorimetry and stoichiometry to measure full body fat and CHO oxidation, whereas we only performed an estimate of resting energy expenditure through a bioimpedance body composition analyser. The whole‐body FAO method used here does not measure oxidation of endogenous fatty acids and is using palmitate as representative for dietary fatty acid oxidation. Thus, this method could be viewed as providing the body a ‘palmitate challenge’ and measuring the response to this fat‐challenge. In this regard, it may be similar to bioenergetics tests where substrate is provided. This study is also limited by its cross‐sectional design, and longitudinal studies will be necessary to determine whether shifts in PBMC bioenergetics precede insulin resistance/dyslipidemia or are an adaptive response to such conditions.

## 5 CONCLUSIONS

To identify bioenergetic alterations associated with obesity and early stages of insulin resistance, we interrogated the bioenergetics of circulating cells and correlations to markers of metabolic health in a cohort of young prepubertal children. PBMCs from children with overweight/obesity exhibit increased mitochondrial respiration, regardless of insulin sensitivity; however, the metabolism of PBMCs from insulin‐resistant children with overweight/obesity is shifted even more towards more mitochondrial respiration and away from glycolysis when compared to PBMCs from insulin‐sensitive overweight/obese children. Platelet bioenergetics were only altered in insulin‐resistant overweight/obese children, with these children having platelets with ~30% less ATP production rates by either mitochondrial respiration or glycolysis, as compared to insulin‐sensitive overweight/obese children. We also report that PBMC bioenergetics correlates reasonably well with whole‐body RMR and FAO, as well as with biomarkers of metabolic health and inflammation. Interestingly, increases in PBMC mitochondrial respiration correlate positively with BMIz, fasting glucose and insulin levels as well as HOMA‐IR, but negatively with inflammatory cytokines. Taken together, our findings are consistent with metabolic adaptations of immune cell metabolism to the metabolic stressors associated with insulin resistance. Further studies are needed to understand whether these metabolic adaptations serve to reduce inflammation and prevent progression to T2D as there may be therapeutic potential in targeting immune cell metabolism in obesity to prevent metabolic decline.

## AUTHOR CONTRIBUTIONS

E.C. and S.R. designed the study and obtained funding. E.B. and M.C. designed the stable isotope/dietary fatty acid oxidation studies. L.M.D. recruited study participants and carried out study visits. S.R. and M.C. processed specimens and conducted the laboratory studies. R.D.L. performed statistical analyses. E.C., S.R. and E.B. interpreted the results. S.R. drafted the manuscript, and all authors revised and edited the manuscript. All authors approve of the final version of the manuscript.

## FUNDING INFORMATION

Research reported in this publication was supported by the Center for Childhood Obesity Prevention and its Scholarly Writing program under Award Number 5P20GM109096 (Arkansas Children's Research Institute, PI: Weber) from the National Institute of General Medical Sciences of the National Institutes of Health, European Union’s Horizon Europe project PAS GRAS ID 101080329. Additional funds were provided by Arkansas Children's Research Institute and Arkansas Biosciences Institute. Investigators were also partly supported by USDA/ARS# 6026‐10700‐001‐000D (EC, MC, EB, SR). EC was also supported by COMPETE 2020 Operational Program for Competitiveness and Internalization and Portuguese national funds via Fundação para a Ciência e a Tecnologia (FCT), I. P, Portugal, under projects POCI‐01‐0145‐FEDER‐007440, UIDB/04539/2020, UIDP/04539/2020, LA/P/0058/2020. The content is solely the responsibility of the authors and does not necessarily represent the official views of the National Institutes of Health or other funders.

## CONFLICT OF INTEREST STATEMENT

The authors declare no conflict of interest, financial or otherwise.

## Supporting information


Appendix S1.

## Data Availability

The data that supports the findings of this study are available in the Appendix S1 of this article.
