# Supplementary material for: Enhanced mitochondrial respiration in peripheral blood mononuclear cells (PBMCs) from young children with overweight/obesity and insulin resistance
Source: Eur J Clin Invest. 2025 Jun 17;55(12):e70090. doi: 10.1111/eci.70090 (PMC12327403; doi:10.1111/eci.70090)
Supplement: Supplementary file 1 — Appendix S1. [file ECI-55-e70090-s001.zip › BMPP SAS code rev.docx]

/*================================================================================

Code prepared by Reid D. Landes, UAMS Biostatistics

rdlandes@uams.edu.

May 2025

010 - Setting options for "Output" window.

- Creating a global variable, LOCATION, from which we will import data and

into which we will export data.

- Importing the raw data.

================================================================================*/

options ls=**100** ps=**55** nodate nonumber formdlim = " " nonotes;

%let LOCATION = /* Your~Location~Here */ ;

**proc** **import** out = WORK1 replace dbms = xlsx

datafile = "&LOCATION\BMPP Raw Data (2024-10-23).xlsx";

sheet = "WORK1";

**run**;

**quit**;

/*================================================================================

011 - METHODS

Study Participants

1st paragraph, 1st sentence.

"A cohort of 65 prepubertal children (5-10 years of age) ... "

================================================================================*/

ods listing select all;

title "Sample size and age range";

**proc** **means** data = WORK1 n min max maxdec=**1**;

var AGE;

**run**;

/*================================================================================

020 - TABLE 1

- Calculating the summary statistics that go into Table 1.

- Formatting those results for an easier-to-read dataset, which we will

export.

================================================================================*/

/* Macro "GetTab1" calculates the summary statistics.

_ORDER - the order of the characteristic as it appears in Table 1

_Y - the characteristic that is being summarized

_ROUNDER - the place to which the summary statistics will be rounded

*/

**%macro** GetTab1( _ORDER , _Y , _ROUNDER);

data TMP100;

set WORK1;

Y = &_Y;

run;

ods listing exclude all;

proc means data = TMP100 n mean stddev ;

var Y;

ods output summary = SUMSTAT0;

run;

data SUMSTAT1;

retain ORDER OUTCOME GROUP Y_MEAN Y_STDDEV;

set SUMSTAT0;

ORDER = &_ORDER;

OUTCOME = "&_Y";

GROUP = "-All-";

run;

proc means data = TMP100 n mean stddev;

class GROUP;

var Y;

ods output summary = SUMSTAT0;

run;

data SUMSTAT2;

retain ORDER OUTCOME GROUP Y_MEAN Y_STDDEV;

set SUMSTAT0;

ORDER = &_ORDER;

OUTCOME = "&_Y";

run;

data SUMSTAT3;

set SUMSTAT1 SUMSTAT2;

MEAN = round(Y_MEAN, &_ROUNDER );

SD = round(Y_StdDev, &_ROUNDER ) ;

N = Y_N;

drop NObs;

run;

proc append data=SUMSTAT3 base = TABLE1_v0 force;

run;

proc datasets;

delete TMP100 SUMSTAT0 - SUMSTAT3;

run;

quit;

**%mend** GetTab1;

/* This is a temporary dataset into which the "GetTab1" macro results will be

stored. */

**data** TABLE1_v0;

input OUTCOME $15. GROUP $5. MEAN SD N ORDER;

cards;

----|----|----|----| -999 -999 -999 -999

;**run**;

/* Calling the macro, "GetTab1" */

%***GetTab1***( **1**, AGE , **0.1**);

%***GetTab1***( **2**, MALE , **0.01**);

%***GetTab1***( **3**, NH_WHITE , **0.01**);

%***GetTab1***( **4**, Height , **0.1**);

%***GetTab1***( **5**, WEIGHT , **0.1**);

%***GetTab1***( **6**, WAIST_CIRC , **0.1**);

%***GetTab1***( **7**, BMIZ , **0.01**);

%***GetTab1***( **8**, SBPz , **0.01**);

%***GetTab1***( **9**, DBPz , **0.01**);

%***GetTab1***( **10**, GLUCOSE , **0.01**);

%***GetTab1***( **11**, INSULIN , **0.01**);

%***GetTab1***( **12**, HOMA_IR , **0.01**);

%***GetTab1***( **13**, Total_Cholesterol , **1**);

%***GetTab1***( **14**, HDL , **0.01**);

%***GetTab1***( **15**, LDL , **0.01**);

%***GetTab1***( **16**, Triglycerides , **0.01**);

%***GetTab1***( **17**, Glycerol , **1**);

%***GetTab1***( **18**, Lactate , **0.01**);

%***GetTab1***( **19**, CRP , **0.01**);

%***GetTab1***( **20**, Leptin , **1**);

%***GetTab1***( **21**, Adiponectin , **1**);

%***GetTab1***( **22**, Leptin_div_Adipo , **0.001**);

%***GetTab1***( **23**, IL6 , **1**);

%***GetTab1***( **24**, MCP1 , **1**);

%***GetTab1***( **25**, TNFalpha , **0.1**);

%***GetTab1***( **26**, IL1beta , **0.1**);

%***GetTab1***( **27**, IL8 , **0.1**);

/* Cleaning up the TABLE1 dataset */

**data** TABLE1;

set TABLE1_V0;

if OUTCOME = "----|----|----|" or GROUP = "----|" then delete;

**run**;

**proc** **sort** data=TABLE1;

by ORDER GROUP;

**run**;

**proc** **datasets**;

delete TABLE1_V0;

**run**;

**quit**;

/* Exporting the TABLE1 dataset */

**proc** **export** data = TABLE1 replace

outfile = "&LOCATION\BMPP Table 1.csv";

**run**;

/*================================================================================

030 - FIGURE 1

- Calculating the summary and inferential statistics that go into Figure 1.

- Formatting those results for an easier-to-read dataset, which we will

export.

================================================================================*/

/* The "OneWay" macro performs a Welch's one-way ANOVA making all 3 pairwise

comparisons between the groups. Normal assumptions are checked. Then we make all

3 pairwise comparisons with Wilcoxon Rank Sum tests.

Y - the outcome that is to be analyzed

_TABLE_ORDER - the order of the outcome as it appears in Table 2

*/

**%macro** OneWay( Y , _TABLE_ORDER);

title "Analyses for &Y";

ods listing exclude all;

proc freq data=WORK1;

where &Y ne **.**;

tables GROUP ;

ods output onewayfreqs = GROUPN0;

run;

data GROUPN1;

set GROUPN0;

N = FREQUENCY;

keep GROUP N;

run;

title2 "1-way ANOVA, group-specific variance";

proc mixed data=WORK1;

class GROUP;

model &Y = GROUP / ddfm=kr2 outp=OUT0;

repeated / group = GROUP;

lsmeans GROUP / pdiff cl adjust = tukey;

ods listing select tests3;

ods output covparms=COVPARM0 lsmeans = LSM0 diffs = DIFF0;

run;

data COVPARM1;

set COVPARM0;

SD = sqrt(ESTIMATE);

GROUP = substr(GROUP, **7**, **8**);

drop COVPARM ESTIMATE;

run;

proc sort data=COVPARM1;

by GROUP;

proc sort data=OUT0;

by GROUP;

data OUT1;

merge OUT0 COVPARM1;

by GROUP;

sRESID = RESID / SD;

run;

proc sort data=GROUPN1;

by GROUP;

proc sort data=COVPARM1;

by GROUP;

proc sort data=LSM0;

by GROUP;

data LSM1;

retain OUTCOME GROUP N MEAN SD LO95 UP95;

merge LSM0 COVPARM1 GROUPN1;

by GROUP;

OUTCOME = "&Y";

MEAN = ESTIMATE;

LO95 = LOWER;

UP95 = UPPER;

TABLE_ORDER = &_TABLE_ORDER;

keep OUTCOME GROUP N MEAN SD LO95 UP95 TABLE_ORDER;

run;

data DIFF1;

retain OUTCOME GROUP _GROUP DIFF StdErr DF PVALUE LO95 UP95 ORDER;

set DIFF0;

OUTCOME = "&Y";

DIFF = ESTIMATE;

PVALUE = PROBT;

LO95 = LOWER;

UP95 = UPPER;

TABLE_ORDER = &_TABLE_ORDER;

keep OUTCOME GROUP _GROUP DIFF StdErr DF PVALUE LO95 UP95 TABLE_ORDER;

run;

title2 "Least Squares Means";

proc print data=LSM1 noobs;

run;

title2 "Differences";

proc print data=DIFF1 noobs;

run;

proc append data=LSM1 base=KEEP_LSM0 force;

proc append data=DIFF1 base=KEEP_DIFF0 force;

run;

title2 "Normality check";

proc univariate data=OUT1 normal plots;

var sRESID;

ods listing select testsfornormality plots;

run;

data TMPWORK1;

set WORK1;

Y = &Y;

run;

proc means data=TMPWORK1 n median q1 q3;

class GROUP;

var Y ;

ods listing exclude summary;

ods output summary = SUMSTAT0;

run;

data SUMSTAT1;

retain OUTCOME GROUP N MEDIAN Q1 Q3;

set SUMSTAT0;

OUTCOME = "&Y";

N = Y_N;

MEDIAN = Y_MEDIAN;

Q1 = Y_Q1;

Q3 = Y_Q3;

TABLE_ORDER = &_TABLE_ORDER;

keep OUTCOME GROUP N MEDIAN Q1 Q3 TABLE_ORDER;

run;

ods listing select none;

proc npar1way data = WORK1;

where GROUP ne "O-IS";

class GROUP;

var &Y;

ods output wilcoxontest = WTEST0;

run;

data WTEST13;

set WTEST0;

GROUP = "N-IS";

_GROUP = "O-IR";

OUTCOME = "&Y";

WilcoxonT_p = nvalue1;

if NAME1 = "PT2_WIL" then output;

keep OUTCOME GROUP _GROUP WilcoxonT_p;

run;

proc npar1way data = WORK1;

where GROUP ne "O-IR";

class GROUP;

var &Y;

ods output wilcoxontest = WTEST0;

run;

data WTEST12;

set WTEST0;

GROUP = "N-IS";

_GROUP = "O-IS";

OUTCOME = "&Y";

WilcoxonT_p = nvalue1;

if NAME1 = "PT2_WIL" then output;

keep OUTCOME GROUP _GROUP WilcoxonT_p;

run;

proc npar1way data = WORK1;

where GROUP ne "N-IS";

class GROUP;

var &Y;

ods output wilcoxontest = WTEST0;

run;

data WTEST23;

set WTEST0;

GROUP = "O-IR";

_GROUP = "O-IS";

OUTCOME = "&Y";

WilcoxonT_p = nvalue1;

if NAME1 = "PT2_WIL" then output;

keep OUTCOME GROUP _GROUP WilcoxonT_p;

run;

data WTEST123;

set WTEST12 WTEST13 WTEST23;

TABLE_ORDER = &_TABLE_ORDER;

run;

ods listing select all;

title2 'Medians & Interquartile Range';

proc print noobs data = SUMSTAT1;

run;

title2 'Wilcoxon Rank Sum Tests';

proc print noobs data = WTEST123;

run;

proc append data=WTEST123 base = KEEP_WTEST0 force;

run;

proc append data=SUMSTAT1 base = KEEP_MED0 force;

run;

proc datasets;

delete OUT0 OUT1 LSM0 LSM1 COVPARM0 COVPARM1 DIFF0 DIFF1 GROUPN0 GROUPN1

TMPWORK1 WTEST0 WTEST12 WTEST13 WTEST23 SUMSTAT0 SUMSTAT1 WTEST123;

run; quit;

**%mend** OneWay;

/* These are temporary datasets into which the "OneWay" macro results will be

stored. */

**data** KEEP_LSM0;

input OUTCOME $20. GROUP $15. N MEAN SD LO95 UP95 TABLE_ORDER;

cards;

----|----|----|----|----|----|----| -999 -999 -999 -999 -999 -999

;**run**;

**data** KEEP_DIFF0;

input OUTCOME $20. GROUP $15. _GROUP $15. DIFF StdErr DF PVALUE LO95 UP95 TABLE_ORDER;

cards;

----|----|----|----|----|----|----|----|----|----| -999 -999 -999 -999 -999 -999 -999

;**run**;

**data** KEEP_WTEST0;

input OUTCOME $20. GROUP $15. _GROUP $15. WilcoxonT_p TABLE_ORDER;

cards;

----|----|----|----|----|----|----|----|----|----| -999 -999

;**run**;

**data** KEEP_MED0;

input OUTCOME $20. GROUP $15. N MEDIAN Q1 Q3 TABLE_ORDER;

cards;

----|----|----|----|----|----|----| -999 -999 -999 -999 -999

;**run**;

/* Calling the "OneWay" macro */

/*~~~TEXT~~~TEXT~~~TEXT~~~TEXT~~~TEXT~~~TEXT~~~TEXT~~~TEXT~~~TEXT~~~TEXT~~~TEXT~~~

Basal respiration was ~25% higher in O-IR as compared to N-IS children (Figure 1A).

~~~TEXT~~~TEXT~~~TEXT~~~TEXT~~~TEXT~~~TEXT~~~TEXT~~~TEXT~~~TEXT~~~TEXT~~~TEXT~~~*/

%***OneWay***( PBMC_BR , **1** );

/*~~~TEXT~~~TEXT~~~TEXT~~~TEXT~~~TEXT~~~TEXT~~~TEXT~~~TEXT~~~TEXT~~~TEXT~~~TEXT~~~

Maximal respiration was 40% and 75% higher in O-IS and O-IR as compared to

N-IS children (Figure 1B)

~~~TEXT~~~TEXT~~~TEXT~~~TEXT~~~TEXT~~~TEXT~~~TEXT~~~TEXT~~~TEXT~~~TEXT~~~TEXT~~~*/

%***OneWay***( PBMC_Max , **2** );

/*~~~TEXT~~~TEXT~~~TEXT~~~TEXT~~~TEXT~~~TEXT~~~TEXT~~~TEXT~~~TEXT~~~TEXT~~~TEXT~~~

...while spare respiratory capacity was 60% and 90% higher in O-IS and O-IR as

compared to N-IS children (Figure 1C).

~~~TEXT~~~TEXT~~~TEXT~~~TEXT~~~TEXT~~~TEXT~~~TEXT~~~TEXT~~~TEXT~~~TEXT~~~TEXT~~~*/

%***OneWay***( PBMC_Spare , **3** );

/*~~~TEXT~~~TEXT~~~TEXT~~~TEXT~~~TEXT~~~TEXT~~~TEXT~~~TEXT~~~TEXT~~~TEXT~~~TEXT~~~

Basal glycolysis rates were not significantly different between groups; (Figure 1D)

~~~TEXT~~~TEXT~~~TEXT~~~TEXT~~~TEXT~~~TEXT~~~TEXT~~~TEXT~~~TEXT~~~TEXT~~~TEXT~~~*/

%***OneWay***( PBMC_BG , **4** );

/*~~~TEXT~~~TEXT~~~TEXT~~~TEXT~~~TEXT~~~TEXT~~~TEXT~~~TEXT~~~TEXT~~~TEXT~~~TEXT~~~

however, OCR/PER, a ratio of mitochondrial respiration to glycolysis, was

significantly lower (e.g. shifted towards glycolysis) in PBMCs from O-IS as

compared to both N-IS and O-IR children (Figure 1E).

~~~TEXT~~~TEXT~~~TEXT~~~TEXT~~~TEXT~~~TEXT~~~TEXT~~~TEXT~~~TEXT~~~TEXT~~~TEXT~~~*/

%***OneWay***( PBMC_OCR_PER , **5** );

/*~~~TEXT~~~TEXT~~~TEXT~~~TEXT~~~TEXT~~~TEXT~~~TEXT~~~TEXT~~~TEXT~~~TEXT~~~TEXT~~~

The rates of ATP production, from either mitochondrial respiration or glycolysis

did not differ significantly between groups (Figure 1F-H).

~~~TEXT~~~TEXT~~~TEXT~~~TEXT~~~TEXT~~~TEXT~~~TEXT~~~TEXT~~~TEXT~~~TEXT~~~TEXT~~~*/

%***OneWay***( PBMC_mitoATP , **6** );

%***OneWay***( PBMC_glycoATP , **7** );

%***OneWay***( PBMC_ATP , **8** );

/* Cleaning up the FIGURE1 datasets; one contains summary statistics, and

the other contains inferential statistics */

**data** TMP10;

input PANEL $1. ;

TABLE_ORDER + **1**;

cards;

A

B

C

D

E

F

G

H

;**run**;

**data** FIGURE1_SumStats1;

set KEEP_LSM0;

if OUTCOME = "----|----|----|----|" then delete;

**proc** **sort**;

by TABLE_ORDER GROUP;

**run**;

**data** FIGURE1_SumStats2;

set KEEP_MED0;

if OUTCOME = "----|----|----|----|" then delete;

**proc** **sort**;

by TABLE_ORDER GROUP;

**run**;

**data** FIGURE1_Sumstat3;

retain TABLE_ORDER;

merge FIGURE1_SumStats1 FIGURE1_SumStats2;

by TABLE_ORDER GROUP;

**run**;

**data** FIGURE1_Sumstat;

retain PANEL;

merge FIGURE1_Sumstat3 TMP10;

by TABLE_ORDER ;

drop TABLE_ORDER ;

**run**;

**data** FIGURE1_InfStat1;

set KEEP_DIFF0;

if OUTCOME = "----|----|----|----|" then delete;

**proc** **sort**;

by TABLE_ORDER GROUP _GROUP;

**run**;

**data** FIGURE1_InfStat2;

set KEEP_WTEST0;

if OUTCOME = "----|----|----|----|" then delete;

**proc** **sort**;

by TABLE_ORDER GROUP _GROUP;

**run**;

**data** FIGURE1_InfStat3;

retain TABLE_ORDER;

merge FIGURE1_InfStat1 FIGURE1_InfStat2;

by TABLE_ORDER GROUP _GROUP;

**run**;

**data** FIGURE1_InfStat;

retain PANEL;

merge FIGURE1_InfStat3 TMP10;

by TABLE_ORDER ;

drop TABLE_ORDER;

**run**;

**proc** **datasets**;

delete FIGURE1_InfStat1 FIGURE1_InfStat2 FIGURE1_InfStat3 FIGURE1_SumStats1 FIGURE1_SumStats2

Figure1_sumstat3 KEEP_WTEST0 KEEP_MED0 KEEP_DIFF0 KEEP_LSM0 TMP10 TMP1 TABLE_v0;

**run**;**quit**;

/* Exporting the FIGURE1 datasets */

**proc** **export** data=FIGURE1_Sumstat replace

outfile = "&LOCATION\BMPP Figure 1 Summary statistics.csv";

**run**;**quit**;

**proc** **export** data=FIGURE1_InfStat replace

outfile = "&LOCATION\BMPP Figure 1 Inferential statistics.csv";

**run**;**quit**;

/*================================================================================

040 - FIGURE 2

- Calculating the summary and inferential statistics that go into Figure 2.

- Formatting those results for an easier-to-read dataset, which we will

export.

================================================================================*/

/* These are temporary datasets into which the "OneWay" macro results will be

stored. */

**data** KEEP_LSM0;

input OUTCOME $20. GROUP $15. N MEAN SD LO95 UP95 TABLE_ORDER;

cards;

----|----|----|----|----|----|----| -999 -999 -999 -999 -999 -999

;**run**;

**data** KEEP_DIFF0;

input OUTCOME $20. GROUP $15. _GROUP $15. DIFF StdErr DF PVALUE LO95 UP95 TABLE_ORDER;

cards;

----|----|----|----|----|----|----|----|----|----| -999 -999 -999 -999 -999 -999 -999

;**run**;

**data** KEEP_WTEST0;

input OUTCOME $20. GROUP $15. _GROUP $15. WilcoxonT_p TABLE_ORDER;

cards;

----|----|----|----|----|----|----|----|----|----| -999 -999

;**run**;

**data** KEEP_MED0;

input OUTCOME $20. GROUP $15. N MEDIAN Q1 Q3 TABLE_ORDER;

cards;

----|----|----|----|----|----|----| -999 -999 -999 -999 -999

;**run**;

/* Calling the "OneWay" macro */

/*~~~TEXT~~~TEXT~~~TEXT~~~TEXT~~~TEXT~~~TEXT~~~TEXT~~~TEXT~~~TEXT~~~TEXT~~~TEXT~~~

When platelet respiration was interrogated using the mitochondrial stress test,

all parameters of mitochondrial respiration were similar between groups

(Figure 2 A-C).

~~~TEXT~~~TEXT~~~TEXT~~~TEXT~~~TEXT~~~TEXT~~~TEXT~~~TEXT~~~TEXT~~~TEXT~~~TEXT~~~*/

%***OneWay***( PLT_BR , **1** );

%***OneWay***( PLT_Max , **2** );

%***OneWay***( PLT_Spare , **3** );

/*~~~TEXT~~~TEXT~~~TEXT~~~TEXT~~~TEXT~~~TEXT~~~TEXT~~~TEXT~~~TEXT~~~TEXT~~~TEXT~~~

While lower rates of glycolysis in platelets from O-IR as compared to O-IS

children (Figure 2D) were observed,

~~~TEXT~~~TEXT~~~TEXT~~~TEXT~~~TEXT~~~TEXT~~~TEXT~~~TEXT~~~TEXT~~~TEXT~~~TEXT~~~*/

%***OneWay***( PLT_BG , **4** );

/*~~~TEXT~~~TEXT~~~TEXT~~~TEXT~~~TEXT~~~TEXT~~~TEXT~~~TEXT~~~TEXT~~~TEXT~~~TEXT~~~

OCR/ECAR did not differ among groups (Figure 2E).

~~~TEXT~~~TEXT~~~TEXT~~~TEXT~~~TEXT~~~TEXT~~~TEXT~~~TEXT~~~TEXT~~~TEXT~~~TEXT~~~*/

%***OneWay***( PLT_OCR_PER , **5** );

/*~~~TEXT~~~TEXT~~~TEXT~~~TEXT~~~TEXT~~~TEXT~~~TEXT~~~TEXT~~~TEXT~~~TEXT~~~TEXT~~~

ATP production rates from both mitochondrial oxidative phosphorylation and

glycolysis were ~30% lower in platelets from O-IR as compared to O-IS

participants (Figure 2F-H).

~~~TEXT~~~TEXT~~~TEXT~~~TEXT~~~TEXT~~~TEXT~~~TEXT~~~TEXT~~~TEXT~~~TEXT~~~TEXT~~~*/

%***OneWay***( PLT_mitoATP , **6** );

%***OneWay***( PLT_glycoATP , **7** );

%***OneWay***( PLT_ATP , **8** );

/* Cleaning up the FIGURE2 datasets; one contains summary statistics, and

the other contains inferential statistics */

**data** TMP10;

input PANEL $1. ;

TABLE_ORDER + **1**;

cards;

A

B

C

D

E

F

G

H

;**run**;

**data** FIGURE2_SumStats1;

set KEEP_LSM0;

if OUTCOME = "----|----|----|----|" then delete;

**proc** **sort**;

by TABLE_ORDER GROUP;

**run**;

**data** FIGURE2_SumStats2;

set KEEP_MED0;

if OUTCOME = "----|----|----|----|" then delete;

**proc** **sort**;

by TABLE_ORDER GROUP;

**run**;

**data** FIGURE2_Sumstat3;

retain TABLE_ORDER;

merge FIGURE2_SumStats1 FIGURE2_SumStats2;

by TABLE_ORDER GROUP;

**run**;

**data** FIGURE2_Sumstat;

retain PANEL;

merge FIGURE2_Sumstat3 TMP10;

by TABLE_ORDER ;

drop TABLE_ORDER ;

**run**;

**data** FIGURE2_InfStat1;

set KEEP_DIFF0;

if OUTCOME = "----|----|----|----|" then delete;

**proc** **sort**;

by TABLE_ORDER GROUP _GROUP;

**run**;

**data** FIGURE2_InfStat2;

set KEEP_WTEST0;

if OUTCOME = "----|----|----|----|" then delete;

**proc** **sort**;

by TABLE_ORDER GROUP _GROUP;

**run**;

**data** FIGURE2_InfStat3;

retain TABLE_ORDER;

merge FIGURE2_InfStat1 FIGURE2_InfStat2;

by TABLE_ORDER GROUP _GROUP;

**run**;

**data** FIGURE2_InfStat;

retain PANEL;

merge FIGURE2_InfStat3 TMP10;

by TABLE_ORDER ;

drop TABLE_ORDER;

**run**;

**proc** **datasets**;

delete FIGURE2_InfStat1 FIGURE2_InfStat2 FIGURE2_InfStat3 FIGURE2_SumStats1 FIGURE2_SumStats2

FIGURE2_SumStat3 KEEP_WTEST0 KEEP_MED0 KEEP_DIFF0 KEEP_LSM0 TMP10;

**run**;**quit**;

/* Exporting the FIGURE2 datasets */

**proc** **export** data=FIGURE2_Sumstat replace

outfile = "&LOCATION\BMPP Figure 2 Summary statistics.csv";

**run**;**quit**;

**proc** **export** data=FIGURE2_InfStat replace

outfile = "&LOCATION\BMPP Figure 2 Inferential statistics.csv";

**run**;**quit**;

/*================================================================================

050 - TABLE 2

- Calculating the summary and inferential statistics that go into Table 2.

- Formatting those results for an easier-to-read dataset, which we will

export.

================================================================================*/

/* These are temporary datasets into which the "OneWay" macro results will be

stored. */

**data** KEEP_LSM0;

input OUTCOME $20. GROUP $15. N MEAN SD LO95 UP95 TABLE_ORDER;

cards;

----|----|----|----|----|----|----| -999 -999 -999 -999 -999 -999

;**run**;

**data** KEEP_DIFF0;

input OUTCOME $20. GROUP $15. _GROUP $15. DIFF StdErr DF PVALUE LO95 UP95 TABLE_ORDER;

cards;

----|----|----|----|----|----|----|----|----|----| -999 -999 -999 -999 -999 -999 -999

;**run**;

**data** KEEP_WTEST0;

input OUTCOME $20. GROUP $15. _GROUP $15. WilcoxonT_p TABLE_ORDER;

cards;

----|----|----|----|----|----|----|----|----|----| -999 -999

;**run**;

**data** KEEP_MED0;

input OUTCOME $20. GROUP $15. N MEDIAN Q1 Q3 TABLE_ORDER;

cards;

----|----|----|----|----|----|----| -999 -999 -999 -999 -999

;**run**;

/* Calling the "OneWay" macro. */

/*~~~TEXT~~~TEXT~~~TEXT~~~TEXT~~~TEXT~~~TEXT~~~TEXT~~~TEXT~~~TEXT~~~TEXT~~~TEXT~~~

RMR differed among all three groups, with N-IS being statistically lower than

O-IS, and O-IS being statistically lower than O-IR.

~~~TEXT~~~TEXT~~~TEXT~~~TEXT~~~TEXT~~~TEXT~~~TEXT~~~TEXT~~~TEXT~~~TEXT~~~TEXT~~~*/

%***OneWay***( RMR , **1** );

/*================================================================================

051 - A special analysis

OneWay ANCOVA of RMR with lean body mass (using fat free mass, FFM) as

a covariate.

"When adjusted for lean body mass, no differences in whole-body fatty acid

oxidation were found among the groups."

================================================================================*/

/*~~~TEXT~~~TEXT~~~TEXT~~~TEXT~~~TEXT~~~TEXT~~~TEXT~~~TEXT~~~TEXT~~~TEXT~~~TEXT~~~

Since RMR can depend on fat free mass, we also used an ANCOVA with fat free mass

as a covariate, and compared the means. In this additional analysis, an increase

of 1 kg in fat free mass was estimated to result in a 31 kcal/day increase in RMR;

however, the ordering of the three groups’ means was the same and the groups still

statistically differed.

~~~TEXT~~~TEXT~~~TEXT~~~TEXT~~~TEXT~~~TEXT~~~TEXT~~~TEXT~~~TEXT~~~TEXT~~~TEXT~~~*/

title "Analyses for RMR";

title2 "1-way ANCOVA, group-specific variance";

title3 "Fat-free mass (FFM) as the covariate";

**proc** **mixed** data=WORK1;

class GROUP;

model RMR = GROUP FFM/ ddfm=kr2 s;

repeated / group = GROUP;

lsmeans GROUP / pdiff cl adjust = tukey;

ods listing select covparms tests3 solutionf lsmeans diffs;

**run**;

/*!@#$%&*!@#$%&*!@#$%&*!@#$%&*!@#$%&*!@#$%&*!@#$%&*!@#$%&*!@#$%&*!@#$%&*!@#$%&*!@#$%&*

/*!@#$%&*!@#$%&*!@#$%&*!@#$%&*!@#$%&*!@#$%&*!@#$%&*!@#$%&*!@#$%&*!@#$%&*!@#$%&*!@#$%&*

/*!@#$%&*!@#$%&*!@#$%&*!@#$%&*!@#$%&*!@#$%&*!@#$%&*!@#$%&*!@#$%&*!@#$%&*!@#$%&*!@#$%&*

data WORKTMP;

retain RandomID GROUP Weight RMR FFM scRMR scFFM BREAK;

set WORK1;

BREAK = "|<<<<<<<<<<<<<<<>>>>>>>>>>>>>>|";

scRMR = RMR / Weight;

FFMpct = 100*FFM / Weight;

run;

title 'Summary stats for scRMR and scFFM';

proc means data = WORKTMP n mean stddev maxdec = 1;

class GROUP;

var scRMR ;

run;

title "Analyses for scRMR";

title2 "1-way ANCOVA, group-specific variance";

title3 " Fat-free mass % (FFMpct) as the covariate";

proc mixed data=WORKTMP;

class GROUP;

model scRMR = GROUP FFMpct/ ddfm=kr2 s;

repeated / group = GROUP;

lsmeans GROUP / pdiff cl adjust = tukey;

ods listing select covparms tests3 solutionf lsmeans diffs;

run;

/*!@#$%&*!@#$%&*!@#$%&*!@#$%&*!@#$%&*!@#$%&*!@#$%&*!@#$%&*!@#$%&*!@#$%&*!@#$%&*!@#$%&*

/*!@#$%&*!@#$%&*!@#$%&*!@#$%&*!@#$%&*!@#$%&*!@#$%&*!@#$%&*!@#$%&*!@#$%&*!@#$%&*!@#$%&*

/*!@#$%&*!@#$%&*!@#$%&*!@#$%&*!@#$%&*!@#$%&*!@#$%&*!@#$%&*!@#$%&*!@#$%&*!@#$%&*!@#$%&*/

/*================================================================================

050 - Continuing with Check 050 above

================================================================================*/

/*~~~TEXT~~~TEXT~~~TEXT~~~TEXT~~~TEXT~~~TEXT~~~TEXT~~~TEXT~~~TEXT~~~TEXT~~~TEXT~~~

The whole-body palmitate oxidation rate of the O-IS group was statistically

higher, by 1.4 times, than that of the N-IS group.

-- SEE CHUNK 51 --

When adjusted for lean body mass, no differences in whole-body fatty acid

oxidation were found among the groups.

~~~TEXT~~~TEXT~~~TEXT~~~TEXT~~~TEXT~~~TEXT~~~TEXT~~~TEXT~~~TEXT~~~TEXT~~~TEXT~~~*/

%***OneWay***( FAO , **2** );

/*~~~TEXT~~~TEXT~~~TEXT~~~TEXT~~~TEXT~~~TEXT~~~TEXT~~~TEXT~~~TEXT~~~TEXT~~~TEXT~~~

Intake of total protein, carbohydrates, fats and total calories did not differ

among groups.

~~~TEXT~~~TEXT~~~TEXT~~~TEXT~~~TEXT~~~TEXT~~~TEXT~~~TEXT~~~TEXT~~~TEXT~~~TEXT~~~*/

%***OneWay***( FAT , **3** );

%***OneWay***( Prot , **4** );

%***OneWay***( Carb , **5** );

%***OneWay***( Cals , **6** );

/*~~~TEXT~~~TEXT~~~TEXT~~~TEXT~~~TEXT~~~TEXT~~~TEXT~~~TEXT~~~TEXT~~~TEXT~~~TEXT~~~

We also tested for differences in general levels of physical activity between

the groups as this may also impact fatty acid oxidation, and based on scores

from the Physical Activity Questionnaire for elementary aged children (PAQ-C),

we did not detect differences between groups in terms of regular physical activity

levels.

~~~TEXT~~~TEXT~~~TEXT~~~TEXT~~~TEXT~~~TEXT~~~TEXT~~~TEXT~~~TEXT~~~TEXT~~~TEXT~~~*/

%***OneWay***( Activity ,**7** );

/* Cleaning up the TABLE2 datasets; one contains summary statistics, and

the other contains inferential statistics */

**data** TABLE2_SumStats1;

set KEEP_LSM0;

if OUTCOME = "----|----|----|----|" then delete;

**proc** **sort**;

by OUTCOME GROUP;

**run**;

**data** TABLE2_SumStats2;

set KEEP_MED0;

if OUTCOME = "----|----|----|----|" then delete;

**proc** **sort**;

by OUTCOME GROUP;

**run**;

**data** TABLE2_Sumstat;

retain TABLE_ORDER;

merge TABLE2_SumStats1 TABLE2_SumStats2;

by OUTCOME GROUP;

**run**;

**data** TABLE2_InfStat1;

set KEEP_DIFF0;

if OUTCOME = "----|----|----|----|" then delete;

**proc** **sort**;

by OUTCOME GROUP _GROUP;

**run**;

**data** TABLE2_InfStat2;

set KEEP_WTEST0;

if OUTCOME = "----|----|----|----|" then delete;

**proc** **sort**;

by OUTCOME GROUP _GROUP;

**run**;

**data** TABLE2_InfStat;

retain TABLE_ORDER;

merge TABLE2_InfStat1 TABLE2_InfStat2;

by OUTCOME GROUP _GROUP;

**run**;

**proc** **datasets**;

delete TABLE2_InfStat1 TABLE2_InfStat2 TABLE2_SumStats1 TABLE2_SumStats2

KEEP_WTEST0 KEEP_MED0 KEEP_DIFF0 KEEP_LSM0;

**run**;**quit**;

/* Exporting the TABLE2 datasets */

**proc** **export** data=TABLE2_SUMSTAT replace

outfile = "&LOCATION\BMPP Table 2 Summary statistics.csv";

**run**;**quit**;

**proc** **export** data=TABLE2_INFSTAT replace

outfile = "&LOCATION\BMPP Table 2 Inferential statistics.csv";

**run**;**quit**;

/*================================================================================

052 - A special analysis

OneWay ANCOVA of FAO with lean body mass (using fat free mass) as

a covariate.

"When adjusted for lean body mass, no differences in whole-body fatty acid

oxidation were found among the groups."

================================================================================*/

title "Analyses for FAO";

title2 "1-way ANCOVA, group-specific variance";

title3 "Fat-free mass (FFM) as the covariate";

**proc** **mixed** data=WORK1;

class GROUP;

model FAO = GROUP FFM/ ddfm=kr2 outp=OUT0;

repeated / group = GROUP;

lsmeans GROUP / pdiff cl adjust = tukey;

ods listing select covparms tests3 lsmeans diffs;

**run**;

/*================================================================================

055 - Gathering p-values from Tables 2 and Figures 1 and 2 so that we can

compute the positive False Discovery Rate (pFDR). pFDR computations are

done in R.

================================================================================*/

**data** TABLE2_TMP1;

retain DISPLAY OUTCOME GROUP _GROUP P;

set TABLE2_INFSTAT;

if OUTCOME = 'RMR' then P = WilcoxonT_p; *Because RMR was not normal;

else P = PVALUE;

DISPLAY = " TABLE 2";

keep DISPLAY OUTCOME GROUP _GROUP P;

**run**;

**data** FIGURE1_TMP1;

retain DISPLAY OUTCOME GROUP _GROUP P;

set FIGURE1_INFSTAT;

DISPLAY = "FIGURE 1";

P = PVALUE;

keep DISPLAY OUTCOME GROUP _GROUP P;

**run**;

**data** FIGURE2_TMP1;

retain DISPLAY OUTCOME GROUP _GROUP P;

set FIGURE2_INFSTAT;

DISPLAY = "FIGURE 2";

P = PVALUE;

keep DISPLAY OUTCOME GROUP _GROUP P;

**run**;

**data** GRP_COMPARE_PVALS;

set FIGURE1_TMP1 FIGURE2_TMP1 TABLE2_TMP1;

**run**;

**proc** **datasets**;

delete FIGURE1_TMP1 FIGURE2_TMP1 TABLE2_TMP1;

**run**;

**quit**;

/* Exporting the GRP_COMPARE_PVALS dataset */

**proc** **export** data=GRP_COMPARE_PVALS replace

outfile = "&LOCATION\BMPP Group comparison p-values for pFDR.csv";

**run**;**quit**;

/*================================================================================

060 - Calculating the summary and inferential statistics that go into Figure 3

and Table 3.

- Formatting those results for an easier-to-read dataset, which we will

export.

================================================================================*/

/* The "GET_CORRS" macro computes the Spearman (or rank) correlations of the two

input variables, X and Y. It also computes 95% confidence intervals for the

estimated correlation.

X - One of the 2 variables to be correlated

Y - The other of the 2 variables to be correlated

_ORDER - the order of the outcome as it appears in Figures and Tables

*/

**%macro** GET_CORRS( X, Y , _ORDER );

ods listing exclude all;

data TMP1;

set WORK1;

X = &X;

Y = &Y;

if X = **.** or Y = **.** then delete;

run;

proc corr data = TMP1 spearman;

var Y; with X;

ods output SpearmanCorr = CORR0 SimpleStats = STATS0;

run;

data STATS1;

set STATS0;

if VARIABLE ne 'X' then delete;

keep VARIABLE NObs;

run;

data CORR1;

retain ORDER X_VAR Y_VAR N CORR PVALUE LOWER UPPER;

merge CORR0 STATS1;

by VARIABLE;

X_VAR = "&X";

Y_VAR = "&Y";

ORDER = &_ORDER;

N = NObs;

CORR = round(Y, **.01**);

PVALUE = round(PY, **.001**);

LOWER = round( tanh( artanh( CORR ) - **1.96** / sqrt( N - **3**) ), **.01**);

UPPER = round( tanh( artanh( CORR ) + **1.96** / sqrt( N - **3**) ), **.01**);

keep ORDER X_VAR Y_VAR N CORR PVALUE LOWER UPPER;

run;

proc append data=CORR1 base=KEEP_CORR0 force;

run;

proc datasets;

delete CORR0 CORR1 STATS0 STATS1 TMP1;

run; quit;

**%mend** GET_CORRS;

/* This is a temporary dataset into which the "GET_CORRS" macro results will be

stored. */

**data** KEEP_CORR0;

input X_VAR $15. Y_VAR $15. N CORR PVALUE LOWER UPPER ORDER;

cards;

----|----|----|----|----|----| -999 -999 -999 -999 -999 -999

; **run**;

/* Calling the "GET_CORRS" macro */

/*~~~TEXT~~~TEXT~~~TEXT~~~TEXT~~~TEXT~~~TEXT~~~TEXT~~~TEXT~~~TEXT~~~TEXT~~~TEXT~~~

Numerous bioenergetics parameters measured in PBMCs correlated positively with

RMR including basal respiration, maximal respiration and spare respiratory

capacity, and also with mitochondrial and total ATP production rates

(Figure 3 A-E).

~~~TEXT~~~TEXT~~~TEXT~~~TEXT~~~TEXT~~~TEXT~~~TEXT~~~TEXT~~~TEXT~~~TEXT~~~TEXT~~~*/

%***GET_CORRS***( RMR , PBMC_BR , **1** ); * Fig 3A;

%***GET_CORRS***( RMR , PBMC_Max , **2** ); * Fig 3B;

%***GET_CORRS***( RMR , PBMC_Spare , **3** ); * Fig 3C;

%***GET_CORRS***( RMR , PBMC_mitoATP , **4** ); * Fig 3D;

%***GET_CORRS***( RMR , PBMC_ATP , **5** ); * Fig 3E;

/*~~~TEXT~~~TEXT~~~TEXT~~~TEXT~~~TEXT~~~TEXT~~~TEXT~~~TEXT~~~TEXT~~~TEXT~~~TEXT~~~

Whole body dietary FAO correlated positively with PBMC spare respiratory capacity

(Figure 3F) and platelet basal glycolysis and glycoATP (Figure 3G-H).

~~~TEXT~~~TEXT~~~TEXT~~~TEXT~~~TEXT~~~TEXT~~~TEXT~~~TEXT~~~TEXT~~~TEXT~~~TEXT~~~*/

%***GET_CORRS***( FAO , PBMC_Spare , **6** ); * Fig 3F;

%***GET_CORRS***( FAO , PLT_BG , **7** ); * Fig 3G;

%***GET_CORRS***( FAO , PLT_glycoATP , **8** ); * Fig 3H;

/* Table 3*/

%***GET_CORRS***( RMR , PBMC_BG , **101**);

%***GET_CORRS***( RMR , PBMC_OCR_PER , **102**);

%***GET_CORRS***( RMR , PBMC_glycoATP , **103**);

/*~~~TEXT~~~TEXT~~~TEXT~~~TEXT~~~TEXT~~~TEXT~~~TEXT~~~TEXT~~~TEXT~~~TEXT~~~TEXT~~~

No significant correlations were found between platelets bioenergetics and RMR.

~~~TEXT~~~TEXT~~~TEXT~~~TEXT~~~TEXT~~~TEXT~~~TEXT~~~TEXT~~~TEXT~~~TEXT~~~TEXT~~~*/

%***GET_CORRS***( RMR , PLT_BR , **104**);

%***GET_CORRS***( RMR , PLT_Max , **105**);

%***GET_CORRS***( RMR , PLT_Spare , **106**);

%***GET_CORRS***( RMR , PLT_BG , **107**);

%***GET_CORRS***( RMR , PLT_OCR_PER , **108**);

%***GET_CORRS***( RMR , PLT_mitoATP , **109**);

%***GET_CORRS***( RMR , PLT_glycoATP , **110**);

%***GET_CORRS***( RMR , PLT_ATP , **111**);

%***GET_CORRS***( FAO , PBMC_BR , **112**);

%***GET_CORRS***( FAO , PBMC_Max , **113**);

%***GET_CORRS***( FAO , PBMC_BG , **114**);

%***GET_CORRS***( FAO , PBMC_OCR_PER , **115**);

%***GET_CORRS***( FAO , PBMC_mitoATP , **116** );

%***GET_CORRS***( FAO , PBMC_glycoATP , **117** );

%***GET_CORRS***( FAO , PBMC_ATP , **118** );

%***GET_CORRS***( FAO , PLT_BR , **119**);

%***GET_CORRS***( FAO , PLT_Max , **120**);

%***GET_CORRS***( FAO , PLT_Spare, **121**);

%***GET_CORRS***( FAO , PLT_OCR_PER , **122**);

%***GET_CORRS***( FAO , PLT_mitoATP , **123** );

%***GET_CORRS***( FAO , PLT_ATP , **124** );

/* Cleaning up the FIGURE3 dataset. */

**data** FIGURE3;

set KEEP_CORR0;

if PVALUE < **0** then delete;

**run**;

**proc** **sort** data=FIGURE3;

by ORDER ;

**run**;

**proc** **datasets**;

delete KEEP_CORR0;

**run**;**quit**;

/* Exporting the FIGURE3 dataset. */

**proc** **export** data = FIGURE3 replace

outfile="&LOCATION\BMPP Figure & Table 3.csv"; **run**;

/*================================================================================

070 - Calculating the summary and inferential statistics that go into Figure 4A.

- Formatting those results for an easier-to-read dataset, which we will

export.

================================================================================*/

/* This is a temporary dataset into which the "GET_CORRS" macro results will be

stored. */

**data** KEEP_CORR0;

input X_VAR $20. Y_VAR $15. N CORR PVALUE LOWER UPPER ORDER;

cards;

----|----|----|----|----|----|----| -999 -999 -999 -999 -999 -999

; **run**;

/*~~~TEXT~~~TEXT~~~TEXT~~~TEXT~~~TEXT~~~TEXT~~~TEXT~~~TEXT~~~TEXT~~~TEXT~~~TEXT~~~

Fasting blood glucose levels positively associated with PBMC basal respiration

(p < 0.001), maximal respiration (p = 0.001) and spare capacity (p = 0.007).

~~~TEXT~~~TEXT~~~TEXT~~~TEXT~~~TEXT~~~TEXT~~~TEXT~~~TEXT~~~TEXT~~~TEXT~~~TEXT~~~*/

%***GET_CORRS***( Glucose , PBMC_BR , **2.1** );

%***GET_CORRS***( Glucose , PBMC_Max , **2.2** );

%***GET_CORRS***( Glucose , PBMC_Spare , **2.3** );

/*~~~TEXT~~~TEXT~~~TEXT~~~TEXT~~~TEXT~~~TEXT~~~TEXT~~~TEXT~~~TEXT~~~TEXT~~~TEXT~~~

Insulin and HOMA-IR both positively associated with PBMC basal respiration

(ps = 0.002 and < 0.001, respectively).

~~~TEXT~~~TEXT~~~TEXT~~~TEXT~~~TEXT~~~TEXT~~~TEXT~~~TEXT~~~TEXT~~~TEXT~~~TEXT~~~*/

%***GET_CORRS***( Insulin , PBMC_BR , **1.1** );

%***GET_CORRS***( HOMA_IR , PBMC_BR , **3.1** );

/*~~~TEXT~~~TEXT~~~TEXT~~~TEXT~~~TEXT~~~TEXT~~~TEXT~~~TEXT~~~TEXT~~~TEXT~~~TEXT~~~

BMIz positively associated with PBMC basal respiration (p = 0.014), maximal

respiration (p = 0.005), and spare capacity (p = 0.007).

~~~TEXT~~~TEXT~~~TEXT~~~TEXT~~~TEXT~~~TEXT~~~TEXT~~~TEXT~~~TEXT~~~TEXT~~~TEXT~~~*/

%***GET_CORRS***( BMIZ , PBMC_BR , **4.1** );

%***GET_CORRS***( BMIZ , PBMC_Max , **4.2** );

%***GET_CORRS***( BMIZ , PBMC_Spare , **4.3** );

/*~~~TEXT~~~TEXT~~~TEXT~~~TEXT~~~TEXT~~~TEXT~~~TEXT~~~TEXT~~~TEXT~~~TEXT~~~TEXT~~~

For blood pressure, SBPz correlated with PBMC basal respiration (p = 0.004),

maximal respiration (p = 0.011), and mitoATP production rate (p = 0.005).

~~~TEXT~~~TEXT~~~TEXT~~~TEXT~~~TEXT~~~TEXT~~~TEXT~~~TEXT~~~TEXT~~~TEXT~~~TEXT~~~*/

%***GET_CORRS***( SBPz , PBMC_BR , **5.1** );

%***GET_CORRS***( SBPz , PBMC_Max , **5.2** );

%***GET_CORRS***( SBPz , PBMC_mitoATP , **5.6** );

/*~~~TEXT~~~TEXT~~~TEXT~~~TEXT~~~TEXT~~~TEXT~~~TEXT~~~TEXT~~~TEXT~~~TEXT~~~TEXT~~~

TNFa negatively correlated with PBMC basal and maximal respiration (p = 0.001

and 0.009, respectively), while IL-6 negatively correlated with basal respiration

(p = 0.004). MCP-1 negatively correlated with PBMC glycolysis (p = 0.008) and

positively correlated with OCR/PER (p=0.005).

~~~TEXT~~~TEXT~~~TEXT~~~TEXT~~~TEXT~~~TEXT~~~TEXT~~~TEXT~~~TEXT~~~TEXT~~~TEXT~~~*/

%***GET_CORRS***( TNFalpha , PBMC_BR , **9.1** );

%***GET_CORRS***( TNFalpha , PBMC_Max , **9.2** );

%***GET_CORRS***( IL6 , PBMC_BR , **7.1** );

%***GET_CORRS***( MCP1 , PBMC_BG , **8.4**);

%***GET_CORRS***( MCP1 , PBMC_OCR_PER , **8.5** );

/*~~~TEXT~~~TEXT~~~TEXT~~~TEXT~~~TEXT~~~TEXT~~~TEXT~~~TEXT~~~TEXT~~~TEXT~~~TEXT~~~

The adipokine leptin correlated positively with PBMC maximal respiration

(p = 0.011) while adiponectin negatively correlated with mitoATP Production rate

(p = 0.008) and the ratio of leptin to adiponectin positively correlated with

PBMC maximal respiration (p = 0.012).

~~~TEXT~~~TEXT~~~TEXT~~~TEXT~~~TEXT~~~TEXT~~~TEXT~~~TEXT~~~TEXT~~~TEXT~~~TEXT~~~*/

%***GET_CORRS***( Leptin , PBMC_Max , **13.2** );

%***GET_CORRS***( Adiponectin , PBMC_mitoATP , **14.6** );

%***GET_CORRS***( Leptin_div_Adipo , PBMC_Max , **15.2** );

/*~~~TEXT~~~TEXT~~~TEXT~~~TEXT~~~TEXT~~~TEXT~~~TEXT~~~TEXT~~~TEXT~~~TEXT~~~TEXT~~~

Among lipids, positive correlations were found between LDL and PBMC maximal

(p = 0.037) and spare capacity (p=026). Interestingly, lactate positively

correlated with basal respiration in PBMCs (p=0.038).

~~~TEXT~~~TEXT~~~TEXT~~~TEXT~~~TEXT~~~TEXT~~~TEXT~~~TEXT~~~TEXT~~~TEXT~~~TEXT~~~*/

%***GET_CORRS***( LDL , PBMC_Max , **17.2** );

%***GET_CORRS***( LDL , PBMC_Spare , **17.3** );

%***GET_CORRS***( Lactate , PBMC_BR , **21.1** );

/* Calling the "GET_CORRS" macro */

%***GET_CORRS***( Insulin , PBMC_Max , **1.2** );

%***GET_CORRS***( Insulin , PBMC_Spare , **1.3** );

%***GET_CORRS***( Insulin , PBMC_BG , **1.4**);

%***GET_CORRS***( Insulin , PBMC_OCR_PER , **1.5** );

%***GET_CORRS***( Insulin , PBMC_mitoATP , **1.6** );

%***GET_CORRS***( Insulin , PBMC_glycoATP , **1.7** );

%***GET_CORRS***( Glucose , PBMC_BG , **2.4**);

%***GET_CORRS***( Glucose , PBMC_OCR_PER , **2.5** );

%***GET_CORRS***( Glucose , PBMC_mitoATP , **2.6** );

%***GET_CORRS***( Glucose , PBMC_glycoATP , **2.7** );

%***GET_CORRS***( HOMA_IR , PBMC_Max , **3.2** );

%***GET_CORRS***( HOMA_IR , PBMC_Spare , **3.3** );

%***GET_CORRS***( HOMA_IR , PBMC_BG , **3.4**);

%***GET_CORRS***( HOMA_IR , PBMC_OCR_PER , **3.5** );

%***GET_CORRS***( HOMA_IR , PBMC_mitoATP , **3.6** );

%***GET_CORRS***( HOMA_IR , PBMC_glycoATP , **3.7** );

%***GET_CORRS***( BMIZ , PBMC_BG , **4.4**);

%***GET_CORRS***( BMIZ , PBMC_OCR_PER , **4.5** );

%***GET_CORRS***( BMIZ , PBMC_mitoATP , **4.6** );

%***GET_CORRS***( BMIZ , PBMC_glycoATP , **4.7** );

%***GET_CORRS***( SBPz , PBMC_Spare , **5.3** );

%***GET_CORRS***( SBPz , PBMC_BG , **5.4**);

%***GET_CORRS***( SBPz , PBMC_OCR_PER , **5.5** );

%***GET_CORRS***( SBPz , PBMC_glycoATP , **5.7** );

%***GET_CORRS***( DBPz , PBMC_BR , **6.1** );

%***GET_CORRS***( DBPz , PBMC_Max , **6.2** );

%***GET_CORRS***( DBPz , PBMC_Spare , **6.3** );

%***GET_CORRS***( DBPz , PBMC_BG , **6.4**);

%***GET_CORRS***( DBPz , PBMC_OCR_PER , **6.5** );

%***GET_CORRS***( DBPz , PBMC_mitoATP , **6.6** );

%***GET_CORRS***( DBPz , PBMC_glycoATP , **6.7** );

%***GET_CORRS***( IL6 , PBMC_Max , **7.2** );

%***GET_CORRS***( IL6 , PBMC_Spare , **7.3** );

%***GET_CORRS***( IL6 , PBMC_BG , **7.4**);

%***GET_CORRS***( IL6 , PBMC_OCR_PER , **7.5** );

%***GET_CORRS***( IL6 , PBMC_mitoATP , **7.6** );

%***GET_CORRS***( IL6 , PBMC_glycoATP , **7.7** );

%***GET_CORRS***( MCP1 , PBMC_BR , **8.1** );

%***GET_CORRS***( MCP1 , PBMC_Max , **8.2** );

%***GET_CORRS***( MCP1 , PBMC_Spare , **8.3** );

%***GET_CORRS***( MCP1 , PBMC_mitoATP , **8.6** );

%***GET_CORRS***( MCP1 , PBMC_glycoATP , **8.7** );

%***GET_CORRS***( TNFalpha , PBMC_Spare , **9.3** );

%***GET_CORRS***( TNFalpha , PBMC_BG , **9.4**);

%***GET_CORRS***( TNFalpha , PBMC_OCR_PER , **9.5** );

%***GET_CORRS***( TNFalpha , PBMC_mitoATP , **9.6** );

%***GET_CORRS***( TNFalpha , PBMC_glycoATP , **9.7** );

%***GET_CORRS***( IL1beta , PBMC_BR , **10.1** );

%***GET_CORRS***( IL1beta , PBMC_Max , **10.2** );

%***GET_CORRS***( IL1beta , PBMC_Spare , **10.3** );

%***GET_CORRS***( IL1beta , PBMC_BG , **10.4**);

%***GET_CORRS***( IL1beta , PBMC_OCR_PER , **10.5** );

%***GET_CORRS***( IL1beta , PBMC_mitoATP , **10.6** );

%***GET_CORRS***( IL1beta , PBMC_glycoATP , **10.7** );

%***GET_CORRS***( IL8 , PBMC_BR , **11.1** );

%***GET_CORRS***( IL8 , PBMC_Max , **11.2** );

%***GET_CORRS***( IL8 , PBMC_Spare , **11.3** );

%***GET_CORRS***( IL8 , PBMC_BG , **11.4**);

%***GET_CORRS***( IL8 , PBMC_OCR_PER , **11.5** );

%***GET_CORRS***( IL8 , PBMC_mitoATP , **11.6** );

%***GET_CORRS***( IL8 , PBMC_glycoATP , **11.7** );

%***GET_CORRS***( CRP , PBMC_BR , **12.1** );

%***GET_CORRS***( CRP , PBMC_Max , **12.2** );

%***GET_CORRS***( CRP , PBMC_Spare , **12.3** );

%***GET_CORRS***( CRP , PBMC_BG , **12.4**);

%***GET_CORRS***( CRP , PBMC_OCR_PER , **12.5** );

%***GET_CORRS***( CRP , PBMC_mitoATP , **12.6** );

%***GET_CORRS***( CRP , PBMC_glycoATP , **12.7** );

%***GET_CORRS***( Leptin , PBMC_BR , **13.1** );

%***GET_CORRS***( Leptin , PBMC_Spare , **13.3** );

%***GET_CORRS***( Leptin , PBMC_BG , **13.4**);

%***GET_CORRS***( Leptin , PBMC_OCR_PER , **13.5** );

%***GET_CORRS***( Leptin , PBMC_mitoATP , **13.6** );

%***GET_CORRS***( Leptin , PBMC_glycoATP , **13.7** );

%***GET_CORRS***( Adiponectin , PBMC_BR , **14.1** );

%***GET_CORRS***( Adiponectin , PBMC_Max , **14.2** );

%***GET_CORRS***( Adiponectin , PBMC_Spare , **14.3** );

%***GET_CORRS***( Adiponectin , PBMC_BG , **14.4**);

%***GET_CORRS***( Adiponectin , PBMC_OCR_PER , **14.5** );

%***GET_CORRS***( Adiponectin , PBMC_glycoATP , **14.7** );

%***GET_CORRS***( Leptin_div_Adipo , PBMC_BR , **15.1** );

%***GET_CORRS***( Leptin_div_Adipo , PBMC_Spare , **15.3** );

%***GET_CORRS***( Leptin_div_Adipo , PBMC_BG , **15.4**);

%***GET_CORRS***( Leptin_div_Adipo , PBMC_OCR_PER , **15.5** );

%***GET_CORRS***( Leptin_div_Adipo , PBMC_mitoATP , **15.6** );

%***GET_CORRS***( Leptin_div_Adipo , PBMC_glycoATP , **15.7** );

%***GET_CORRS***( HDL , PBMC_BR , **16.1** );

%***GET_CORRS***( HDL , PBMC_Max , **16.2** );

%***GET_CORRS***( HDL , PBMC_Spare , **16.3** );

%***GET_CORRS***( HDL , PBMC_BG , **16.4**);

%***GET_CORRS***( HDL , PBMC_OCR_PER , **16.5** );

%***GET_CORRS***( HDL , PBMC_mitoATP , **16.6** );

%***GET_CORRS***( HDL , PBMC_glycoATP , **16.7** );

%***GET_CORRS***( LDL , PBMC_BR , **17.1** );

%***GET_CORRS***( LDL , PBMC_BG , **17.4**);

%***GET_CORRS***( LDL , PBMC_OCR_PER , **17.5** );

%***GET_CORRS***( LDL , PBMC_mitoATP , **17.6** );

%***GET_CORRS***( LDL , PBMC_glycoATP , **17.7** );

%***GET_CORRS***( Triglycerides , PBMC_BR , **18.1** );

%***GET_CORRS***( Triglycerides , PBMC_Max , **18.2** );

%***GET_CORRS***( Triglycerides , PBMC_Spare , **18.3** );

%***GET_CORRS***( Triglycerides , PBMC_BG , **18.4**);

%***GET_CORRS***( Triglycerides , PBMC_OCR_PER , **18.5** );

%***GET_CORRS***( Triglycerides , PBMC_mitoATP , **18.6** );

%***GET_CORRS***( Triglycerides , PBMC_glycoATP , **18.7** );

%***GET_CORRS***( Total_Cholesterol , PBMC_BR , **19.1** );

%***GET_CORRS***( Total_Cholesterol , PBMC_Max , **19.2** );

%***GET_CORRS***( Total_Cholesterol , PBMC_Spare , **19.3** );

%***GET_CORRS***( Total_Cholesterol , PBMC_BG , **19.4**);

%***GET_CORRS***( Total_Cholesterol , PBMC_OCR_PER , **19.5** );

%***GET_CORRS***( Total_Cholesterol , PBMC_mitoATP , **19.6** );

%***GET_CORRS***( Total_Cholesterol , PBMC_glycoATP , **19.7** );

%***GET_CORRS***( Glycerol , PBMC_BR , **20.1** );

%***GET_CORRS***( Glycerol , PBMC_Max , **20.2** );

%***GET_CORRS***( Glycerol , PBMC_Spare , **20.3** );

%***GET_CORRS***( Glycerol , PBMC_BG , **20.4**);

%***GET_CORRS***( Glycerol , PBMC_OCR_PER , **20.5** );

%***GET_CORRS***( Glycerol , PBMC_mitoATP , **20.6** );

%***GET_CORRS***( Glycerol , PBMC_glycoATP , **20.7** );

%***GET_CORRS***( Lactate , PBMC_Max , **21.2** );

%***GET_CORRS***( Lactate , PBMC_Spare , **21.3** );

%***GET_CORRS***( Lactate , PBMC_BG , **21.4**);

%***GET_CORRS***( Lactate , PBMC_OCR_PER , **21.5** );

%***GET_CORRS***( Lactate , PBMC_mitoATP , **21.6** );

%***GET_CORRS***( Lactate , PBMC_glycoATP , **21.7** );

/* Cleaning up the FIGURE4A dataset. */

**data** FIGURE4A;

set KEEP_CORR0;

if PVALUE < **0** then delete;

ROW_COL = ORDER;

drop ORDER;

**run**;

**proc** **sort** data=FIGURE4A;

by ROW_COL ;

**run**;

**proc** **datasets**;

delete KEEP_CORR0;

**run**;

**quit**;

/* Exporting the FIGURE4A dataset. */

**proc** **export** data = FIGURE4A replace

outfile="&LOCATION\BMPP Figure 4A.csv";

**run**;

/*================================================================================

080 - Calculating the summary and inferential statistics that go into Figure 4B.

- Formatting those results for an easier-to-read dataset, which we will

export.

================================================================================*/

/* This is a temporary dataset into which the "GET_CORRS" macro results will be

stored. */

**data** KEEP_CORR0;

input X_VAR $20. Y_VAR $15. N CORR PVALUE LOWER UPPER ORDER;

cards;

----|----|----|----|----|----|----| -999 -999 -999 -999 -999 -999

; **run**;

/*~~~TEXT~~~TEXT~~~TEXT~~~TEXT~~~TEXT~~~TEXT~~~TEXT~~~TEXT~~~TEXT~~~TEXT~~~TEXT~~~

Insulin and HOMA-IR both correlated negatively with platelet glycolysis

(ps = 0.017 and 0.038, respectively) while DBPz negatively correlated with

platelet spare respiratory capacity (p = 0.009).

~~~TEXT~~~TEXT~~~TEXT~~~TEXT~~~TEXT~~~TEXT~~~TEXT~~~TEXT~~~TEXT~~~TEXT~~~TEXT~~~*/

%***GET_CORRS***( Insulin , PLT_BG , **1.4**);

%***GET_CORRS***( HOMA_IR , PLT_BG , **3.4**);

%***GET_CORRS***( DBPz , PLT_Spare , **6.3** );

/*~~~TEXT~~~TEXT~~~TEXT~~~TEXT~~~TEXT~~~TEXT~~~TEXT~~~TEXT~~~TEXT~~~TEXT~~~TEXT~~~

Among markers of inflammation, IL6 and TNFa were found to correlate positively

with platelet spare capacity respiration (ps = 0.010 and 0.040, respectively),

while IL1ß was negatively correlated with platelet OCR/PER (p=0.039).

~~~TEXT~~~TEXT~~~TEXT~~~TEXT~~~TEXT~~~TEXT~~~TEXT~~~TEXT~~~TEXT~~~TEXT~~~TEXT~~~*/

%***GET_CORRS***( IL6 , PLT_Spare , **7.3** );

%***GET_CORRS***( TNFalpha , PLT_Spare , **9.3** );

%***GET_CORRS***( IL1beta , PLT_OCR_PER , **10.5** );

/*~~~TEXT~~~TEXT~~~TEXT~~~TEXT~~~TEXT~~~TEXT~~~TEXT~~~TEXT~~~TEXT~~~TEXT~~~TEXT~~~

Among the lipids measured, total cholesterol correlated positively with

platelet basal respiration (p = 0.047).

~~~TEXT~~~TEXT~~~TEXT~~~TEXT~~~TEXT~~~TEXT~~~TEXT~~~TEXT~~~TEXT~~~TEXT~~~TEXT~~~*/

%***GET_CORRS***( Total_Cholesterol , PLT_BR , **19.1** );

/* Calling the "GET_CORRS" macro */

%***GET_CORRS***( Insulin , PLT_BR , **1.1** );

%***GET_CORRS***( Insulin , PLT_Max , **1.2** );

%***GET_CORRS***( Insulin , PLT_Spare , **1.3** );

%***GET_CORRS***( Insulin , PLT_OCR_PER , **1.5** );

%***GET_CORRS***( Insulin , PLT_mitoATP , **1.6** );

%***GET_CORRS***( Insulin , PLT_glycoATP , **1.7** );

%***GET_CORRS***( Glucose , PLT_BR , **2.1** );

%***GET_CORRS***( Glucose , PLT_Max , **2.2** );

%***GET_CORRS***( Glucose , PLT_Spare , **2.3** );

%***GET_CORRS***( Glucose , PLT_BG , **2.4**);

%***GET_CORRS***( Glucose , PLT_OCR_PER , **2.5** );

%***GET_CORRS***( Glucose , PLT_mitoATP , **2.6** );

%***GET_CORRS***( Glucose , PLT_glycoATP , **2.7** );

%***GET_CORRS***( HOMA_IR , PLT_BR , **3.1** );

%***GET_CORRS***( HOMA_IR , PLT_Max , **3.2** );

%***GET_CORRS***( HOMA_IR , PLT_Spare , **3.3** );

%***GET_CORRS***( HOMA_IR , PLT_OCR_PER , **3.5** );

%***GET_CORRS***( HOMA_IR , PLT_mitoATP , **3.6** );

%***GET_CORRS***( HOMA_IR , PLT_glycoATP , **3.7** );

%***GET_CORRS***( BMIZ , PLT_BR , **4.1** );

%***GET_CORRS***( BMIZ , PLT_Max , **4.2** );

%***GET_CORRS***( BMIZ , PLT_Spare , **4.3** );

%***GET_CORRS***( BMIZ , PLT_BG , **4.4**);

%***GET_CORRS***( BMIZ , PLT_OCR_PER , **4.5** );

%***GET_CORRS***( BMIZ , PLT_mitoATP , **4.6** );

%***GET_CORRS***( BMIZ , PLT_glycoATP , **4.7** );

%***GET_CORRS***( SBPz , PLT_BR , **5.1** );

%***GET_CORRS***( SBPz , PLT_Max , **5.2** );

%***GET_CORRS***( SBPz , PLT_Spare , **5.3** );

%***GET_CORRS***( SBPz , PLT_BG , **5.4**);

%***GET_CORRS***( SBPz , PLT_OCR_PER , **5.5** );

%***GET_CORRS***( SBPz , PLT_mitoATP , **5.6** );

%***GET_CORRS***( SBPz , PLT_glycoATP , **5.7** );

%***GET_CORRS***( DBPz , PLT_BR , **6.1** );

%***GET_CORRS***( DBPz , PLT_Max , **6.2** );

%***GET_CORRS***( DBPz , PLT_BG , **6.4**);

%***GET_CORRS***( DBPz , PLT_OCR_PER , **6.5** );

%***GET_CORRS***( DBPz , PLT_mitoATP , **6.6** );

%***GET_CORRS***( DBPz , PLT_glycoATP , **6.7** );

/* */

%***GET_CORRS***( IL6 , PLT_BR , **7.1** );

%***GET_CORRS***( IL6 , PLT_Max , **7.2** );

%***GET_CORRS***( IL6 , PLT_BG , **7.4**);

%***GET_CORRS***( IL6 , PLT_OCR_PER , **7.5** );

%***GET_CORRS***( IL6 , PLT_mitoATP , **7.6** );

%***GET_CORRS***( IL6 , PLT_glycoATP , **7.7** );

%***GET_CORRS***( MCP1 , PLT_BR , **8.1** );

%***GET_CORRS***( MCP1 , PLT_Max , **8.2** );

%***GET_CORRS***( MCP1 , PLT_Spare , **8.3** );

%***GET_CORRS***( MCP1 , PLT_BG , **8.4**);

%***GET_CORRS***( MCP1 , PLT_OCR_PER , **8.5** );

%***GET_CORRS***( MCP1 , PLT_mitoATP , **8.6** );

%***GET_CORRS***( MCP1 , PLT_glycoATP , **8.7** );

%***GET_CORRS***( TNFalpha , PLT_BR , **9.1** );

%***GET_CORRS***( TNFalpha , PLT_Max , **9.2** );

%***GET_CORRS***( TNFalpha , PLT_BG , **9.4**);

%***GET_CORRS***( TNFalpha , PLT_OCR_PER , **9.5** );

%***GET_CORRS***( TNFalpha , PLT_mitoATP , **9.6** );

%***GET_CORRS***( TNFalpha , PLT_glycoATP , **9.7** );

/* */

%***GET_CORRS***( IL1beta , PLT_BR , **10.1** );

%***GET_CORRS***( IL1beta , PLT_Max , **10.2** );

%***GET_CORRS***( IL1beta , PLT_Spare , **10.3** );

%***GET_CORRS***( IL1beta , PLT_BG , **10.4**);

%***GET_CORRS***( IL1beta , PLT_mitoATP , **10.6** );

%***GET_CORRS***( IL1beta , PLT_glycoATP , **10.7** );

%***GET_CORRS***( IL8 , PLT_BR , **11.1** );

%***GET_CORRS***( IL8 , PLT_Max , **11.2** );

%***GET_CORRS***( IL8 , PLT_Spare , **11.3** );

%***GET_CORRS***( IL8 , PLT_BG , **11.4**);

%***GET_CORRS***( IL8 , PLT_OCR_PER , **11.5** );

%***GET_CORRS***( IL8 , PLT_mitoATP , **11.6** );

%***GET_CORRS***( IL8 , PLT_glycoATP , **11.7** );

%***GET_CORRS***( CRP , PLT_BR , **12.1** );

%***GET_CORRS***( CRP , PLT_Max , **12.2** );

%***GET_CORRS***( CRP , PLT_Spare , **12.3** );

%***GET_CORRS***( CRP , PLT_BG , **12.4**);

%***GET_CORRS***( CRP , PLT_OCR_PER , **12.5** );

%***GET_CORRS***( CRP , PLT_mitoATP , **12.6** );

%***GET_CORRS***( CRP , PLT_glycoATP , **12.7** );

%***GET_CORRS***( Leptin , PLT_BR , **13.1** );

%***GET_CORRS***( Leptin , PLT_Max , **13.2** );

%***GET_CORRS***( Leptin , PLT_Spare , **13.3** );

%***GET_CORRS***( Leptin , PLT_BG , **13.4**);

%***GET_CORRS***( Leptin , PLT_OCR_PER , **13.5** );

%***GET_CORRS***( Leptin , PLT_mitoATP , **13.6** );

%***GET_CORRS***( Leptin , PLT_glycoATP , **13.7** );

%***GET_CORRS***( Adiponectin , PLT_BR , **14.1** );

%***GET_CORRS***( Adiponectin , PLT_Max , **14.2** );

%***GET_CORRS***( Adiponectin , PLT_Spare , **14.3** );

%***GET_CORRS***( Adiponectin , PLT_BG , **14.4**);

%***GET_CORRS***( Adiponectin , PLT_OCR_PER , **14.5** );

%***GET_CORRS***( Adiponectin , PLT_mitoATP , **14.6** );

%***GET_CORRS***( Adiponectin , PLT_glycoATP , **14.7** );

%***GET_CORRS***( Leptin_div_Adipo , PLT_BR , **15.1** );

%***GET_CORRS***( Leptin_div_Adipo , PLT_Max , **15.2** );

%***GET_CORRS***( Leptin_div_Adipo , PLT_Spare , **15.3** );

%***GET_CORRS***( Leptin_div_Adipo , PLT_BG , **15.4**);

%***GET_CORRS***( Leptin_div_Adipo , PLT_OCR_PER , **15.5** );

%***GET_CORRS***( Leptin_div_Adipo , PLT_mitoATP , **15.6** );

%***GET_CORRS***( Leptin_div_Adipo , PLT_glycoATP , **15.7** );

%***GET_CORRS***( HDL , PLT_BR , **16.1** );

%***GET_CORRS***( HDL , PLT_Max , **16.2** );

%***GET_CORRS***( HDL , PLT_Spare , **16.3** );

%***GET_CORRS***( HDL , PLT_BG , **16.4**);

%***GET_CORRS***( HDL , PLT_OCR_PER , **16.5** );

%***GET_CORRS***( HDL , PLT_mitoATP , **16.6** );

%***GET_CORRS***( HDL , PLT_glycoATP , **16.7** );

%***GET_CORRS***( LDL , PLT_BR , **17.1** );

%***GET_CORRS***( LDL , PLT_Max , **17.2** );

%***GET_CORRS***( LDL , PLT_Spare , **17.3** );

%***GET_CORRS***( LDL , PLT_BG , **17.4**);

%***GET_CORRS***( LDL , PLT_OCR_PER , **17.5** );

%***GET_CORRS***( LDL , PLT_mitoATP , **17.6** );

%***GET_CORRS***( LDL , PLT_glycoATP , **17.7** );

%***GET_CORRS***( Triglycerides , PLT_BR , **18.1** );

%***GET_CORRS***( Triglycerides , PLT_Max , **18.2** );

%***GET_CORRS***( Triglycerides , PLT_Spare , **18.3** );

%***GET_CORRS***( Triglycerides , PLT_BG , **18.4**);

%***GET_CORRS***( Triglycerides , PLT_OCR_PER , **18.5** );

%***GET_CORRS***( Triglycerides , PLT_mitoATP , **18.6** );

%***GET_CORRS***( Triglycerides , PLT_glycoATP , **18.7** );

%***GET_CORRS***( Total_Cholesterol , PLT_Max , **19.2** );

%***GET_CORRS***( Total_Cholesterol , PLT_Spare , **19.3** );

%***GET_CORRS***( Total_Cholesterol , PLT_BG , **19.4**);

%***GET_CORRS***( Total_Cholesterol , PLT_OCR_PER , **19.5** );

%***GET_CORRS***( Total_Cholesterol , PLT_mitoATP , **19.6** );

%***GET_CORRS***( Total_Cholesterol , PLT_glycoATP , **19.7** );

%***GET_CORRS***( Glycerol , PLT_BR , **20.1** );

%***GET_CORRS***( Glycerol , PLT_Max , **20.2** );

%***GET_CORRS***( Glycerol , PLT_Spare , **20.3** );

%***GET_CORRS***( Glycerol , PLT_BG , **20.4**);

%***GET_CORRS***( Glycerol , PLT_OCR_PER , **20.5** );

%***GET_CORRS***( Glycerol , PLT_mitoATP , **20.6** );

%***GET_CORRS***( Glycerol , PLT_glycoATP , **20.7** );

%***GET_CORRS***( Lactate , PLT_BR , **21.1** );

%***GET_CORRS***( Lactate , PLT_Max , **21.2** );

%***GET_CORRS***( Lactate , PLT_Spare , **21.3** );

%***GET_CORRS***( Lactate , PLT_BG , **21.4**);

%***GET_CORRS***( Lactate , PLT_OCR_PER , **21.5** );

%***GET_CORRS***( Lactate , PLT_mitoATP , **21.6** );

%***GET_CORRS***( Lactate , PLT_glycoATP , **21.7** );

/* Cleaning up the FIGURE4B dataset. */

**data** FIGURE4B;

set KEEP_CORR0;

if PVALUE < **0** then delete;

ROW_COL = ORDER;

drop ORDER;

**run**;

**proc** **sort** data=FIGURE4B;

by ROW_COL ;

**run**;

**proc** **datasets**;

delete KEEP_CORR0;

**run**;

**quit**;

/* Exporting the FIGURE4A dataset. */

**proc** **export** data = FIGURE4B replace

outfile="&LOCATION\BMPP Figure 4B.csv";

**run**;

/*================================================================================

095 - Gathering p-values from Table 3 and Figures 3 and 4 so that we can

compute the positive False Discovery Rate (pFDR). pFDR computations are

done in R.

================================================================================*/

**data** FIGURE3_TMP1;

set FIGURE3;

DISPLAY = " TABLE 3";

P = PVALUE;

keep DIPLAY X_VAR Y_VAR P;

**run**;

**data** FIGURE4_TMP1;

set FIGURE4A;

DISPLAY = "FIGURE 4A";

P = PVALUE;

keep DIPLAY X_VAR Y_VAR P;

**run**;

**data** FIGURE4_TMP2;

set FIGURE4B;

DISPLAY = "FIGURE 4B";

P = PVALUE;

keep DIPLAY X_VAR Y_VAR P;

**run**;

**data** CORR_PVALS;

set FIGURE3_TMP1 FIGURE4_TMP1 FIGURE4_TMP2;

**run**;

**proc** **datasets**;

delete FIGURE3_TMP1 FIGURE4_TMP1 FIGURE4_TMP2;

**run**;

**quit**;

/* Exporting the CORR_PVALS dataset */

**proc** **export** data=CORR_PVALS replace

outfile = "&LOCATION\BMPP Correlation p-values for pFDR.csv";

**run**;**quit**;
